# Supplementary material for: Improving quality and use of routine health information system data in low- and middle-income countries: A scoping review
Source: PLoS One. 2020 Oct 8;15(10):e0239683. doi: 10.1371/journal.pone.0239683 (PMC7544093; doi:10.1371/journal.pone.0239683)
Supplement: S1 Box — A. Combinations of search terms formulated in Ovid for studies on data quality interventions. B. Combinations of search terms formulated in Ovid for studies on data use interventions. C. Contents of the data charting format used. (DOCX) [file pone.0239683.s001.docx]

**S1A Box: Combinations of search terms formulated in Ovid for studies on data quality interventions**

| 1. Information system* or health information system* or integrated advanced information management system* or management information systems* or medical records system* .ti, ab, kw. OR 2. Clinical laboratory information system* or clinical pharmacy information system* or hospital information system* .ti, ab, kw. AND 3. health plan implementation* or health services research* or regional health planning* or capacity building* or decision making* or health facility administration* or hospital administration* or knowledge management* or management audit* or management information system* or organizational culture* or patient identification system* or public health administration* or records as topic* or total quality management* or guideline adherence* or quality assurance* or quality improvement* or quality indicators, health care* .ti, ab, kw. 4. Data accuracy .ti, ab, kw. Or 5. Data quality .ti, ab, kw. AND 6. LMIC (using an OVID filter list of countries) 7. 1 or 2 8. 4 or 5 9. 3 AND 7 AND 8 AND 6 |
| --- |

**S1B Box: Combination of search terms formulated on Ovid for studies on data use intervention**

| 1. information system* or health information system* or integrated advanced information management system* or management information systems* or medical records system*.ti, ab, kw. OR 2. Clinical laboratory information system* or clinical pharmacy information system* or hospital information system* .ti, ab, kw. AND 3. health plan implementation* or health services research* or regional health planning* or capacity building* or decision making* or health facility administration* or hospital administration* or knowledge management* or management audit* or management information system* or organizational culture* or patient identification system* or public health administration* or records as topic* or total quality management* or guideline adherence* or quality assurance* or quality improvement* or quality indicators, health care* .ti, ab, kw. 4. health information exchange* or decision making, computer-assisted* or data interpretation, statistical* or "information storage and retrieval" or health information interoperability* or public health informatics* .ti, ab, Kw. 5. LMIC (using an OVID filter list of countries) 6. 1 or 2; 7. 6 AND 3 AND 4 AND 5 |
| --- |

**S1C Box: Contents of the data charting format used**

| 1. Publication characteristics: title, year of publication, author, design, study setting, population 2. Phenomena under study (intervention):    1. Type of intervention evaluated or described    2. Target population for the intervention    3. Effect of the intervention    4. Opportunities or barriers to performance of the intervention    5. Outcome expected to change    6. Contextual factors that influenced the outcome 3. Overall conclusions by the authors 4. Limitation of the study reported by the authors |
| --- |
