# Supplementary material for: Improving quality and use of routine health information system data in low- and middle-income countries: A scoping review
Source: PLoS One. 2020 Oct 8;15(10):e0239683. doi: 10.1371/journal.pone.0239683 (PMC7544093; doi:10.1371/journal.pone.0239683)
Supplement: S1 Table — A. Characteristics of studies on data quality interventions in low- and middle-income countries 2008–2020. B. Characteristics of studies on data use interventions in low- and middle-income countries 2008–2020. (DOCX) [file pone.0239683.s002.docx]

**S1A Table: Characteristics of studies on data quality interventions in low and middle-income countries 2008-2020**

| **Author** | **Design** | **Country** | **Intervention** | **Population** | **Outcome** | **Result of intervention** |
| --- | --- | --- | --- | --- | --- | --- |
| Maokola, W., 2011 (29) | Survey | Tanzania | Enhancement of the HMIS: Personal digital assistance (PDA) based system in all health centres. Training and assignment of PDA operator, monthly supervision, on the job training for health center staffs, discuss on monthly generated reports. | 6 hospitals, 13 health centres, and 149 dispensaries | Data quality completeness and timeliness | Incompleteness, 41% of blood slide readings lacked a corresponding out-patient department record. |
| Braa, J., 2011 (30) | Evaluation design, implementation research | Zanzibar | Quarterly data-use workshops for district health management team members, each for five days, systematic peer review, stimulating self-assessment and identification of common issues relating to data quality. | District health management team | Data quality and use | Data collection forms were simplified, duplication of data collection eliminated, data quality improved because of increased use of quality checks at the facility level and by districts. Increased use of indicators at local levels and analysis. |
| Monda J., 2012 (31) | Case study | Kenya | Developed a data integrity module, a plug-in to identify, enumerate, and facilitate correction of errors within an e-health system. Academic Model Providing Access to Healthcare called AMPATH Medical Record System | Clinics, 27 parent and 31 satellite clinics for HIV-treatment | Data quality | This tool uncovered thousands of records with errors and provided direct links to these records to allow for easy correction of identified mistakes. |
| Dombrowsk JG., 2015 (45) | Document review | Brazil, one remote northern region | Live birth information system comparing quality and coverage of Live Births Information System with data from the Civil Registry of the Brazilian Institute of Geography and Statistics. | Live birth information system vs. civil registry 2005 and 2011 | Data quality: incomplete-ness in coverage | Coverage has shown reduction compared to civil registration and incompleteness. 11/15 variables have shown increased incompleteness between 2005 and 2011. |
| Tuti T., 2016 (33) | Case study | Kenya, | Improving routine clinical documentation. Feedback and mentorship through telephone calls and four monthly face-to-face meetings. A non-commercial electronic tool, research electronic data capture (REDCap) was used to enter data and to check data quality. R software auto-generated code was used for running on-site checks. | Hospitals and clinicians treating paediatric in-patients | Data quality | Clinical documentation had improved, the median rate of missing clinical documentation had shown a decline. |
| Mutale W., 2013 (25) | Case study | Ghana, three districts,  Mozambique, one district,  Rwanda, three districts  Tanzania, three districts and  Zambia, some rural health districts | Ghana: A simplified information capturing system as part of District health information management system-2, district health planning and reporting toolkit (to identify and allocate resources based on the district level burden of disease profile)  Mozambique: Providing ongoing feedback on missing data and outliers, and ongoing data quality assessment at all level. Training, support of personnel to monitor the performance of HIS, timely feedback and annual data quality assessment  Rwanda: Quarterly data quality audit and automated data quality report based on logic error generated when administrative and clinical reports are developed  Tanzania: Facility supervisors review Community Health Agent’s (CHA) reports and provide feedback. Health information summary forms were developed to record aggregate data and report each month. CHAs and supervisors meet regularly to review monthly outputs, identify and troubleshoot problems, and plan jointly with the health system. Project coordinators, district coordinators, and supervisors hold similar meetings quarterly and transfer CHA health information to district and project managers for planning and program improvement  Zambia: Standardized protocols for data capture with a real-time query of data gaps. Follow-up during monitoring visits, use of an electronic data capture system and mobile technology to improve quality of data captured | District health management team and health facilities  Mozambique: Health system; health facility, district, and province  Rwanda: health centres  Tanzania: Health workers in the community, dispensary, health centre and hospital  Zambia: clinics | Data quality and use | Ghana: A simplified register was piloted. Its physical size was reduced, which reduced documentation burden and added time for service. Streamlining data collection and aggregation operations, simplifying and computerising feedback to view data feedback and compare performance with counterparts.  Mozambique: Piloted and refined a province-specific data quality assessment methodology. Annual assessment results are disseminated using a simplified ranking system. Use of data has improved. Periodic district-level review and planning meetings promote active data review  Rwanda: Enhanced Electronic Medical Record (EMR), training for data officers and coordinators on quarterly basis, formal and on the job training for clinicians  Tanzania: Adapted and adopted existing community level health information data capture tools, integrated community level data with the routine health information system facility and district level, however, didn’t facilitate data use at Community Health Agent’s level as the system lacks household and client information.  Zambia: Trained 72 clinic supporters as data clerks, five reports were generated, reviewing and clarifying data entry fields reduced the data entry workload. Computers with low-power requirements prevented disruption. Clinic performance reports available at the clinic level. Patient-level information was used. |
| Mpofu M., 2014 (32) | Evaluation design, qualitative | Botswana, 10 districts | New university graduates with no health background recruited and trained as monitoring & evaluation officers and deployed in each district, to manage health related data at district. | District monitoring & evaluation officers | Data quality, management and reporting | Improved district level capacity to monitor and evaluate programs, improved data quality, management and reporting (accuracy, timeliness, validity and completeness). |
| Gimbel S., 2017 (34) | Mixed-methods comparative study | Mozambique, one province,  Rwanda, two districts and  Zambia, three districts | Mozambique: Annual data quality assessments carried out. Written feedback provided to all district and facility managers via a simplified summary data quality ranking tool. Data dashboard to simplify data visualization. Targeted supportive supervision for sites with weaker performance on data quality. Monitoring and evaluation training; district performance review and enhancement meetings.  Rwanda: Enhanced Electronic Medical Records. Data Quality Audits. Community-level data assessed quarterly for concordance and completeness. Assessment of the effect of supporting enhanced supervision and mentorship. Annual consistency and internal validity assessment; monthly data review meetings between project and district health staff.  Zambia: Promoting completeness of clinical forms, expanded electronic medical record (EMR) system, data quality assessment system for Community health information system. Continuous, on-site mentoring, community outreach. Active data collection at the household level, patient follow up and referral system by health workers. | Districts, health facilities and health workers and managers at all level. | Data quality | Mozambique: Substantial improvements in data availability (84% to 99%) and consistency (54% to 87%) led to targeted resource allocation, including intensive training and supervision of weaker performing facilities and districts.  Rwanda: Improved linkages between facility and community health programs, reliable, real-time data collected by Community Health Workers, informed health system planning. Countrywide adoption of some of the project activities such as auditing.  Zambia: A strengthened link between Community Health Workers and Ministry of Health staffs. Improved participation of health facility personnel in Quality Improvement work, including data quality checks. Community Data Quality Assessment created bi-directional feedback mechanism between facilities and communities |
| Ismail S., 2017 (42) | Evaluation | Pakistan, six urban clinics | Granular data model (Maternal and child health Record system). Design and development of a standard-compliant data access model for maintaining maternal and child health data. | Maternal and child health | Improved record-keeping and inter-operability | Health care providers and the majority of women understood all functionalities.  Many said it was effective in improving record keeping. Decreased the burden of creating two records one for a patient and the other for health facility. Most patients regard it as useful. Several doctors are willing to use it. |
| Puttkammer N., 2017 (46) | Case study | Haiti, Port-au-Prince | Identify automated data queries, inspired by the Kahn framework, for a large-scale multi-site Electronic Medical Record System. Comparing two strategies, a Delphi strategy where participants provided input during multiple cycles and reacted to group responses from each prior cycle for HIV-care, and a Burden-of-Disease strategy to establish priories for data quality assurance. | Site managers, clinicians, and disease reporting officers from hospitals and clinics | Data quality | Data quality quires prioritized were, completeness, timeliness and accuracy and internal consistency. With each domain area, 5–15 data quality indicators were identified. |
| Rado R., 2018 (35) | Evaluation design, mixed methods | Five regions in Southern Madagascar | Reinforced integrated disease surveillance and response where data was transferred using SMS. | 294 health care structures in 18 districts | Data quality, | In the last ten SMS, 47% no missing observations. The mean number of erroneous data transmitted were 12 (0-51). Data completeness in the last 4 weeks were 73% and timeliness 43%. |
| Trumbo SP., 2018 (47) | Mixed methods | Mexico and Peru. Two national registers | Electronic immunization register.  Mexico: Computer-based that registered immunization status of children and pregnant women.  Peru: Census database of children <6 years of age; added to collect immunization data, then nutrition. Provided incentive to promote the use of census data base in Peru. | Health facilities, documents, stakeholders | Data quality | Mexico: Poor data recording practice.  Peru: Better data quality, saved time through the use of a single data entry point for different intervention. |
| Ndira SP., 2008 (36) | Mixed-methods, pre-post test | Uganda. Maternal and Child Health-department of one district hospital | Electronic Health Management Information System. Comparison of paper-based reporting and electronic system for routine health data. | Health system as a whole. But one district was selected for the study | Data quality | Availability of reports improved from 79% to 100%. Timeliness was improved, 13 out of 19 were in time at end line. Just an hour was needed to do compilation and reporting. Accuracy level without stillbirth was 82%. However, difficult to establish a statistical association for the difference in accuracy pre-post. |
| Kaushik A., 2015 (43) | Case study | India, the State of Tamil Nadu | Enterprise architecture for e-health web-based HMIS solution, migration from paper-based to an electronic health record, ensure that the integration of institutional and personal health information and medical records is managed effectively, deliver measurable cost and quality results from improved information management in healthcare, and convert granular data to information for decision making. | 276 secondary-care hospitals, 1539 primary healthcare centres, 46 tertiary care institutions and 19 medical colleges | Data quality and data use | The Tamil Nadu HMIS provided critical health data across the health chain for quick and timely intervention by health directorates. Captured data from both facility-level records and administrative sources. Improved resources planning, improved supply chain management of essential drugs, built a robust equipment management system to manage and monitor the functioning of critical equipment and reduced downtime through systematic tracking. |
| Wagenaar BH., 2017 (37) | Qualitative study | Mozambique,  Rwanda and  Zambia | Mozambique: Modified Plan-Do-Study- Act framework. Plan: identify data quality problems, implement facility-based Data quality assessments, training of sub-national managers on data analysis and output presentation approach. Do: data review and feedback meeting, data-driven action plan and supervision and follow-up on the action plan.  Rwanda: Mentoring on Data quality assessment. Plan: Training on data review, quality improvement and analysis. Do: integrate data driven performance review at different level, capacity building on monitoring and evaluation using RHIS.  Zambia: Plan: Create new tools for data entry, visualisation and analysis, pre-implementation facility assessment, training. Do: upgrade the infrastructure, clinic support worker tasks, use new data tools. | Principal investigators of each project, key program implementers and Ministry of Health. | Data quality and use | Data concordance reached >80%, significant increase in data quality, across all sites <10% to >80%. Mozambique and Rwanda managed to sustain data-driven decision making and scale it up to other places. All countries adopted iterative performance measurement and feedback. All teams achieved at least stage IV or V on the modified Berwick's data utilization stage for quality improvement. |
| O'Connor, EC., 2019 (39) | Cluster randomized trial | Sierra Leone | Participatory community-based health information system (PCBHIS). Community health review meetings, monthly Community health workers reports from the intervention communities, facilitate the holding of bimonthly community health data review meetings to share local surveillance data and verbal autopsy information in the intervention communities. | Community health workers | Data use and quality | Community health workers submit monthly reports significantly more frequently to their peer supervisor. Use of health information in planning showed increment in the intervention group but it was not statistically significant. Review of and contribution to community health workers’ activity plans did not show statistically significant increment. |
| Lazzerini, M., 2019 (44) | Observational | Sri Lanka, De Soysa Maternity Hospital | Individual patient data base in hospitals | Hospitals, delivery and new born care data | Data quality and use | Data completeness exceeded other existing hospital recording systems. Less than 1% data were missing for maternal variables and less than 3% for new born variables. Mistakes in data collection and entry occurred in 0.01% and 0.09% of maternal and new born data, respectively. Data analyses and results were reported to the local coordinator and decision makers. Service quality problems were identified for decision making. |
| Njuguna,C., 2019 (40) | Case study | Sierra Leone | Development of customized guidelines, phased training of IDSR focal person, distribution of data collection and reporting tools. Periodic supportive supervision and data quality assessments. Rapid response teams were formed to investigate and respond to disease outbreak alerts in all districts. | Health workers, clinicians from both private and public | Data quality: timely reporting | Annual average proportion of timely weekly reporting by health facility were 93% in 2016 and 97% in 2017. Suspected outbreaks and public health events detected were 96% in 2016 and 100% in 2017. Shift from the paper-based reporting to electronic surveillance system. Use of weekly epidemiological bulletins as a feedback mechanism. |
| Nakibuuka, J., 2019 (38) | Intervention | Uganda, one district | An Unstructured Supplementary Service Data (USSD)-based health data reporting intervention. | Health facilities | Data quality | A total of 224 reports were submitted over the two-month study period. Of the submitted reports, 76.3% were complete compared to required 100%. 71.9%) were accurate, and 70.5% of reports submitted on time. The deficiencies were largely attributed to a few facilities, as only 17.9% of facilities had data discrepancies, 96.4% of the facilities had complete reports and 87.4% of the facilities reported on time. |
| Yourkavitch, J., 2019 (41) | Mixed method | Democratic republic of Congo (DRC),  Malawi, Mozambique,  Niger,  Nigeria, Abia state, and  Nigeria, Niger state | Data quality assessment done twice in 15-month interval, each assessment followed by identification of problem and action. | Community health workers | Data quality | DRC: Stronger results in all but one domain; CHWs were comfortable using reporting forms.  Malawi: High scores in each domain: -Trainings for CHWs and supervisors were conducted.  Mozambique: integrated Community Case Management (iCCM) data reporting system well-established; all but one domain scored highly; regular CHW supervision visits occurred.  Niger: System assessment yielded moderate scores.  Nigeria, Abia state: High scores except for one domain; but errors in aggregated data at supervisors’ level were seen.  written guidance for data reporting was not available.  Nigeria, Niger state: Moderate scores but CHWs did not use data well. |
|  |  |  | DRC: Modified data collection tool  Malawi: Community health workers (CHW) and supervisors training on data collection and reporting, preparation of job aid for CHW on reporting.  Mozambique: Monitoring & Evaluation manual updated including data management.  Niger: Training of staff at all level on reporting, registers revised to facilitate completeness.  Nigeria, Abia state: Refresher trainings for Community health workers (CHW) and supervisors, developed summary reporting form to aggregate data from CHWs and promote supervision of CHWs; held regular data review meetings.  Nigeria, Niger state: Refresher training for supervisors, processes to improve data use and quality established. |  |  |  |

**S1B Table: Characteristics of literature on data use interventions in low- and middle-income countries 2008-2020**

| **Author** | **Design** | **Country** | **Intervention** | **Population** | **Outcome** | **Result of intervention** |
| --- | --- | --- | --- | --- | --- | --- |
| Gaumer G., 2008 (48) | Case study | Egypt, Suez governorate | Feedback and Analytic Comparison Tool (FACT), identified quality problems and the gap in service use by patients. An information system development as part of health sector reform in Egypt | Primary care clinics | Data use | Local authorities found FACT useful, and clinicians have begun to use the feedback function to review and improve practice patterns. Adoption was fast |
| Ndira SP., 2008 (36) | Mixed-methods, pre-post test | Uganda, Maternal and Child Health-department of one district hospital | Comparison of paper-based reporting and electronic system for reporting routine health data | Health system as a whole. But one district in Uganda was selected for the study | Data quality and availability | Availability of reports improved from 79% to 100%. Timeliness was improved, 13 out of 19 were in time at end line. Accuracy level without stillbirth was 82%. However, difficult to establish a statistical association for the difference in accuracy pre-post |
| Braa J., 2012 (30) | Evaluation design, implementation research | Zanzibar | Quarterly data-use workshops for district health management team members, each for five days.  Systematic peer review, stimulating self-assessment and identification of common issues relating to data quality | District health management team | Data quality and use | Simplified forms, duplication of data collection eliminated. Data quality improved, use of computer checks by districts and practical experience gained under supervision during workshops. Increased use of indicators at local levels and the analysis of coverage and quality of service delivery. |
| Matheson AL., 2012 (58) | Case study | Haiti, HIV care and treatment clinics | iSanté, an electronic medical record specifically for HIV care clinics | Hospitals where HIV care is provided | Data use | 67 sites adopted iSanté using different workflows to combine the paper and computerized systems with varying scope. A different level of data use for the clinical purpose. There was a consistent gap between the number of sites with iSanté installed and those using it |
| La Vincente S., 2013 (55) | Case study | Philippines, three local government units | Investment case approach. A decision-support model that estimates the cost and impact of alternative maternal neonatal and child health approaches was used to guide the selection and prioritisation of strategies and rounds of workshops on problem identification. | Three selected sub-national units, two rural provinces and one urbanised city | Data use for planning intervention | Local officials found the structured analysis of health system constraints helpful to identify problems and select locally appropriate strategies |
| Nutlay T., 2013 (50) | Qualitative assessment | Kenya, six districts, rural and urban | District Health Profile, a decision-support system applied at the district level. An excel-based system linked with routine data from health facilities. 11 questions including one on data quality, included a standardised denominator and a target for activities | Health facilities | Data use | Improved data analysis, review, interpretation and sharing. Faster data sharing, reduced error, improved data quality through ease of visualising data quality problems. Improved data use for decision making allowed and motivated immediate response to observed problems in the health system |
| Mutale W., 2013 (25) | Case study | Ghana, three districts,  Mozambique, one district,  Rwanda, three districts  Tanzania, three districts and  Zambia, some rural health districts | Ghana: Simplified information capturing system as part of District health information management system-2. District health planning and reporting toolkit (to identify and allocate resources based on the district level burden of disease profile)  Mozambique: Providing ongoing feedback on missing data and outliers, and ongoing data quality assessment at all levels.  Activities include training, and support to personnel to monitor the performance of the health information system, and timely feedback and annual data quality assessment  Rwanda: Quarterly data quality audit and automated data quality report based on logic error generated when administrative and clinical reports are developed | Ghana: District health management team and health facilities  Mozambique: Health system; health facility, district, and province  Rwanda: 33 health centres  Tanzania: Health workers in the community, dispensary, health centre and hospital  Zambia: clinics | Data quality and use | Ghana: A simplified register was piloted. Its physical size was reduced, reduced documentation burden and additional time for service. Streamlined data collection and aggregation operations, simplified and computerized feedback to view data feedback and compare performance with counterparts  Mozambique: Piloted and refined a province-specific data quality assessment methodology.  Annual assessment results were disseminated using a simplified ranking system. Use of data had improved. Periodic district-level review and planning meetings promoted active data review  Rwanda: Enhanced electronic medical record, training for data officers and coordinators on quarterly basis, formal and on the job training for clinicians. |
|  |  |  | Tanzania: Facility supervisors review Community Health Agent’s (CHA) reports and provide feedback. Health information summary forms were developed to record aggregate data and report each month. CHAs and supervisors meet regularly to review monthly outputs, identify and troubleshoot problems, and plan jointly with the health system. Project coordinators, district coordinators, and supervisors hold similar meetings quarterly and transfer CHA health information to district and project managers for planning and program improvement.  Zambia: Standardized protocols for data capture with a real-time query of data gaps. Follow-up during monitoring visits, use of an electronic data capture system and mobile technology to improve quality of data captured |  |  | Tanzania: Adapted and adopted existing community level health information data capture tools, integrated community level data with the routine health information system facility and district level, however, didn’t facilitate data use at Community Health Agent’s level as the system lacks household and client information  Zambia: Trained 72 clinic supporters as data clerks, five reports were generated, reviewing and clarifying data entry fields reduced the data entry workload. Computers with low-power requirements prevented disruption.  Clinic performance reports available at the clinic level. Patient-level information was used |
| Hosseini M., 2014 (56) | Case study | Iran, Alzahra Education and Treatment Centre | Developing an immunization information system that is interoperable based-on service-oriented architecture and health level 7 | 1500 immunization records from 400 infants, a sample of 50 infants were checked in both immunization information system | Data use | The intervention allowed the two systems in heterogeneous platforms and architectures to negotiate and interoperate with web services, without any need to change their architecture. 100 % accuracy between the two immunization information systems |
| Rajan D., 2014 (52) | Case study | DR Congo, a model, a construct of a district with 10 health centres and one primary referral hospital | Resource planning model based on WHO Integrated Health Care Technology package. Created a database of 200 clinical and non-clinical interventions and over 2000 procedures. Each intervention breaks down to procedures and, then to resource needed for each procedure. Salaries and price of each were entered into the software and database. This calculation answers how much is necessary per inhabitant and year to operationally run a health centre | Health system | Use of data for decision | Results were used as a negotiation and advocacy instrument at negotiations with the ministry of finance and when submitting successful proposals to secure funding. Allowed Ministry of Health (MOH) to better negotiate with donors and stakeholders. Used as an instrument to align donors, managed to channel a large amount of funds to field activities instead of administration and field planning. Assisted MOH and others to plan their district health service, helped do other costing exercises at national and district level. |
| Kaushik A., 2015 (43) | Case study | India, the State of Tamil Nadu | Enterprise architecture for e-health web-based HMIS solution, migration from paper-based to an electronic health record, ensure that the integration of institutional and personal health information and medical records is managed effectively, deliver measurable cost and quality results from improved information management in healthcare, and convert granular data to information for decision making | 276 secondary-care hospitals, 1539 primary healthcare centres, 46 tertiary care institutions and 19 medical colleges | Data quality and data use | The Tamil Nadu HMIS provided critical health data across the health chain for quick and timely intervention by health directorates. Captured data from both facility-level records and administrative sources. Improved resources planning, improved supply chain management of essential drugs, built a robust equipment management system to manage and monitor the functioning of critical equipment and reduced downtime through systematic tracking |
| Landis-Lewis Z., 2015 (53) | Qualitative assessment | Malawi, 8 out of 66 existing facilities providing ART for HIV | Electronic medical record in ART clinics. Understanding barriers to individualized performance feedback using e-Health data by interviews, observations and informant feedback | ART clinics and health care providers | Use of data for feedback | Four key barriers to the use of electronic medical record data were identified; rotation of staff, disruption of care processes, user acceptance and lifespan of indicators |
| Wagenaar BH., 2017 (37) | Qualitative study | Mozambique  Rwanda  Zambia | Mozambique: Modified Plan-Do-Study-Act (PDSA) framework. Plan: Identify data quality problems, implement facility-based data quality assessments, training of sub-national managers on data analysis and output presentation approach. Do: Data review and feedback meeting, data-driven action plan and supervision and follow-up on the action plan  Rwanda: Mentoring on Data quality assessment. Plan: Training on data review, quality improvement and analysis. Do: integrate data driven performance review at different level, capacity building on monitoring and evaluation using RHIS  Zambia: Plan: Create new tools for data entry, visualisation and analysis, pre-implementation facility assessment, training. Do: upgrade the infrastructure, clinic support worker tasks, use new data tools | Principal investigators of each project, key program implementers as well as Ministry of Health counterparts. | Data quality and use | Data concordance reached >80%, significant increase in data quality, across all sites <10% to >80%. Mozambique and Rwanda managed to sustain data-driven decision making and scale it up to other places. All countries adopted iterative performance measurement and feedback. All teams achieved at least stage IV or V on the modified Berwick's data utilization stage for quality improvement. |
| Uneke JC., 2019 (54) | Mixed methods | Nigeria | Five phases to develop policy information platform (PIP). Created a PIP website where policy-relevant documents and scientific papers were uploaded. The website became official. Developed a PIP with 412 published articles, policy docs and grey information | Policymakers | Data use | Access and efficiency, allows easy access to articles relevant for research. Policy-relevant evidence, the local evidence available on the web was useful. Networking and collaboration fostered exchanges and inter-linkages related to evidence-informed decision making. Strengthened capacities in use of data for decision making. User-friendly platform. Strong acceptability and feasibility |
| Beane, A., 2019 (57) | Case study | Sri Lanka | Clinician-led, acute care digital mHealth platform | Hospitals | Availability of data and use for decision in patient care | The digital intensive care unit platform increased availability of variables for benchmarking acuity of admissions and supported the validation of prognostic models. Overcame existing burden of collecting and reporting manually. Mobile version of the digital platform empowered nurses and doctors to adopt digital vital sign reporting in busy ward settings |
| O'Connor, EC., 2019 (39) | Cluster randomized trial | Sierra Leone | Participatory community-based health information system (PCBHIS). Community health review meetings, monthly Community health workers reports from the intervention communities, facilitate the holding of bimonthly Community Health Data Review Meetings to share local surveillance data and verbal autopsy information in the intervention communities (every two months) | Community health workers | Data quality and use | Community health workers submit monthly reports significantly more frequently to their peer supervisor. Use of health information in planning showed increment in the intervention group but it was not statistically significant. Review of and contribution to Community health workers activity plans did not show statistically significant increment |
| Lazzerini, M., 2019 (44) | Observational | Sri Lanka, De Soysa Maternity Hospital | Individual patient data base in hospitals | Hospitals; delivery and new born care data | Data quality and use | Data completeness exceeded other existing hospital recording systems. Less than 1% data were missing for maternal variables and less than 3% for new-born variables. Mistakes in data collection and entry occurred in 0.01% and 0.09% of maternal and new born data, respectively.  Data analysed and results were reported to local coordinator and disseminated to decision makers, service quality problems were identified for decision making. |

*iSanté: a multi-site electronic medical record implemented by the Haitian Ministry of Health.
