# Supplementary material for: Improving quality and use of routine health information system data in low- and middle-income countries: A scoping review
Source: PLoS One. 2020 Oct 8;15(10):e0239683. doi: 10.1371/journal.pone.0239683 (PMC7544093; doi:10.1371/journal.pone.0239683)
Supplement: S1 File — (DOCX) [file pone.0239683.s003.docx]

**Protocol**

**Improving Quality of Routine Health Information System and Data Use in Low and Middle Income Countries: A Scoping Review**

**Prepared by: Seblewengel Lemma,**

**Reviewed by: Lars-Åke Persson, Deepthi Wickremasinghe, Carina Källestål**

**Project Name: Operational Research and Coaching for analysts,**

**Place: Ethiopia**

**Date: 2018**

**Table of Contents**

[**Acronyms** 3](#_Toc536612127)

[**Summary** 4](#_Toc536612128)

[**Background** 5](#_Toc536612129)

[**Objective:** 6](#_Toc536612130)

[**Methodology** 7](#_Toc536612131)

[**Reference** 12](#_Toc536612132)

# **Acronyms**

DAC: Development and Assistant Committee
DQR: Data Quality Review

EPHI: Ethiopian Public Health Institute

FMOH: Federal Ministry of Health Ethiopia

HIS: Health Information system

ORCA: Operational Research and Coaching for Analyst

PFSA: Pharmaceutical Funding and Supply Agency

RHIS: Routine Health Information System

WHO: World Health Organization

# **Summary**

**Background:** A routine health information system is one of the essential components of a health system. Interventions have been conducted to improve routine data quality and use for decision-making in low and middle-income countries. These interventions differ in design, methods, and scope. So far, there have been limited efforts to synthesize the available knowledge across the currently available intervention studies from low- and middle-income countries.

**Objective:** To review and synthesize published results from interventions that aimed at improving data quality and use in routine health information systems in low and middle income countries.

**Method:** This scoping review will include intervention studies that aimed to improve data quality and use within routine health information systems in low- and middle-income countries, published in English from 2008 to 2018. Literature will be searched in the databases Medline/PubMed, Web of Science, and Scopus based on predefined combinations of search terms. Additional search will be conducted to update the literature search later in the process. Two reviewers will identify study reports and extract data based on a set of criteria using a pretested data extraction form. Narrative analysis will be conducted to present the results.

# **Background**

Health Information System (HIS) is one of the six building blocks of a health system designed to meet information needs within the health system. It generates health information vital for planning, monitoring and evaluation of public health programs and interventions (1–3). Decisions are made all the time and everywhere in the health system and information generated in the system influences decisions from patient care, to policy formation and implementation, thereby influencing the health in a community (1).

The HIS generates information mainly from the routine health information system (RHIS), which is health service based data and the population based data, coming from: surveys, census and vital event registrations(4). HIS performance is measured based on the quality of these generated data and their use for decision making (1).

Thus, quality data are vital for the health system to function well and for policy makers to evaluate the effect of their efforts to improve the health of the population (2). The quality of RHIS data have improved across the globe (5,6), but health systems in low-and-middle income countries are still suffering from suboptimal quality and use of data generated by their routine health information systems (7–12). The data quality issues have often been expressed as incomplete registers (13,14), lack of consistency between registers and reports (10,11,15,16), and or low level of data accuracy (12). Discrepancies have often been noted between results from data generated in the routine health information system and population-based surveys (14).

Despite the increasing availability of health information, the use of such information for decision making is still poor in many low-and-middle income countries (17). Studies from these settings show low or inadequate use of data, especially of routinely generated data (18–21). Studies in health facilities or based on interviews with health workers have frequently reported low use of such data for planning in spite of collecting, aggregating, and generating data reports to the next level of the health system ( 18,20,21). This limited use of data may also partly be explained by the lack of trust in the quality of data (17,20).

Given the vital role of RHIS in the health system, there have been a number of interventions that have tried to improve the quality and use of data (8,22–25). The design, methods, and scope of these interventions vary, however, making it difficult to draw general conclusions on which interventions could be successful in what contexts.

The London School of Hygiene and Tropical Medicine(LSHTM) is implementing an intervention project called Operational Research and Coaching for Analyst (ORCA) in collaboration with the Ethiopian Public Health Institute and the Federal Ministry of Health, to contribute to the improvement of data quality and use in the Ethiopian routine health information system. This scoping review is part of the ORCA initiative, designed to map and synthesis results from such interventions on routine data quality and use.

# **Objective:**

To review and synthesize published results from interventions that aimed at improving data quality and use in routine health information systems in low and middle income countries.

**Research question:**

- Which interventions have successfully improved RHIS data quality and use in low- and middle-income countries, and what contextual factors have influenced the outcome?

# **Methodology**

**Study eligibility criteria.** Individual studies will be selected based on the following inclusion criteria (see table 1 for the exclusion criteria):

**Participants:** This review will consider studies published in peer reviewed scientific journals which are conducted across all or parts of the health system in low and middle-income countries that describe or assess interventions or approaches used to improve the health workers’ or health care facilities’ contribution and performance so that data quality and use are strengthened.

**Study design and period:** this review considers studies of any design with the aim of evaluating or describing a given intervention intended to improve data quality or use. Qualitative studies, which assess potential barriers and facilitators for the success of such interventions in the health information system, will also be included. The systematic review will consider articles published from 2008 to 2018.

**Phenomena of interest:** this review will focus on interventions implemented to improve data quality issues of RHIS as well as its use by health workers and decision makers at different levels of a given health system. Data quality is defined based on its attributes or dimensions, and these dimensions vary depending on different tools used. For instance, the WHO data quality review tool defines routine data quality as data completeness, timeliness, consistency, and accuracy (26). In the routine data quality assessment tool prepared by MEASURE Evaluation, the data quality dimensions are grouped into two dimensions as main and sub-dimensions; the main dimension consists of accuracy, and reliability and the sub-dimensions are precision, completeness, timeliness, integrity and confidentiality (27).

This review will also look at any potential interventions conducted and tested to improve data use in the RHIS. Data use is defined as the use of RHIS data for decision making at any level of a health system. We will look for studies, which reported use of such interventions or evaluate them.

**Language:** All studies published in English will be included.

**Publication status:** This review will cover only published articles. (see Table 1)

Table 1: Summary of inclusion and Exclusion Criteria

| Inclusion criteria | Exclusion criteria |
| --- | --- |
| - Studies published in peer-reviewed scientific journals of any design meant to evaluate or describe interventions used to improve data quality or use of data from routine health information system | - Studies not focusing on routine health information system - Grey literature - Systematic reviews |
| - Studies published in English | - Language other than English |
| - Studies published from January 2008 to 2018 | - Studies published before January 2008 |
| - Studies conducted in low- and middle-income countries according to the Development Assistance Committee (DAC)* list, 2018 | - Studies conducted in high-income countries |

**Information source and search strategy:**

The following bibliographic data-bases will be searched for literature: Medline/PubMed, Web of Science, global health and Embase based on predefined combination of search terms. Available published studies’ reference lists will also be reviewed to ensure saturation. A combination of search terms, prepared based on the objective of this scoping review will be evaluated by a librarian to make sure the combination of the search terms can answer our objectives.

The following are proposed combinations of search terms formulated on Ovid:

1. Information system* or health information system* or routine health information system* or integrated advanced information management system* or management information system* or medical records system .ti, ab, kw.
2. Clinical laboratory information system* or clinical pharmacy information system* or hospital information system* or district health information system or patient identification system .ti, ab, kw.
3. health plan implementation or health services research* or regional health planning or capacity building or decision making or health facility administration or hospital administration or knowledge management or management audit or organi#ational culture * or public health administration or records as topic* or total quality management or guideline adherence or quality assurance or quality improvement or quality indicators, health care .ti, ab, kw.
4. Data accuracy* or data valid* or data reliability* or data consistency* or data complete* or data quality* or data error .ti, ab, kw. Or
5. LMIC (a list of countries from OVID filter was used)
6. 1 or 2
7. 4 or 5
8. 3 AND 7 AND 8 AND 6

For the second objective:

1. Information system* or health information system* or routine health information system* or integrated advanced information management system* or management information system* or medical records system* .ti, ab, kw.
2. Clinical laboratory information system* or clinical pharmacy information system* or hospital information system* or patient identification system* or district health information system .ti, ab, kw.
3. health plan implementation or health services research* or regional health planning or capacity building or decision making or health facility administration* or hospital administration* or knowledge management* or management audit* or organi#ational culture* or public health administration or records as topic* or total quality management or guideline adherence or quality assurance or quality improvement or quality indicators, health care* .ti, ab, kw.
4. health information exchange* or decision making, data-informed or data interpretation, statistical or "information storage and retrieval" or health information interoperability or public health informatics or information use* .ti, ab, kw.
5. LMIC (a list of countries from OVID filter was used)
6. 1 or 2
7. 6 AND 3 AND 4 AND 5

Literature search will be limited to studies conducted from 2008 to 2018; additional search will be done to update the search at some point in the future.

The retrieved studies will be exported to Zotero reference manager, where duplicates will be managed. First, two reviewers (SL and AJ) will screen the title, then the abstract of the articles based on eligibility criteria set above; this process will be done on Zotero. Second, once the articles are selected based on their abstract, the reviewers will thoroughly review the full articles to determine those that meet the inclusion criteria and decide whether the study should be included or not. In case where they disagree, the third reviewer CK will review the articles and give the final decision. One of the reviewers (SL) will go through the reference list of the selected articles to ensure saturation.

**Data Extraction:**

Once the articles are confirmed for inclusion in the review, the list will be exported in to excel. The first two reviewers (SL and AJ) will extract data from each article based on the following points:

1. Publication characteristics: title, year of publication, author, design, study setting, population
2. Phenomena under study (intervention):
   1. Type of intervention evaluated or described
   2. Target population for the intervention
   3. Effect of the intervention
   4. Opportunities or barriers to performance of the intervention
   5. Outcome expected to change
   6. Contextual factors that influenced the outcome
3. Overall conclusions by the authors
4. Limitation of the study reported by the authors

This data extraction form will be tested and will be modified if need be. If further information is needed, authors of individual articles will also be contacted.

**Synthesis:**

A narrative analysis will be conducted to present the results of this scoping review. The presentation of the results will follow the checklist for reporting of scoping review “Preferred Reporting Items for Systematic Reviews and Meta-Analysis: extension for Scoping Reviews (PRISMA-ScR)”(28). The analysis will include: a description of the literature search, and tables will be used to summarize characteristics of the articles and their results. The analysis will summarize the type of interventions used to improve data quality and use, and intervention effect will be summarized if reported in the individual articles. The analysis will identify potential challenges of such interventions, and contextual factors that may influence the outcome in low-and-middle income countries. Finally, lessons learnt from the described interventions will be summarized.

# **Reference**

1. Strengthening Health Information Systems in Low- and Middle-Income Countries—A Model to Frame What We Know and What We Need to Learn — MEASURE Evaluation [Internet]. [cited 2019 Jan 24]. Available from: https://www.measureevaluation.org/resources/publications/tr-17-156

2. AbouZahr C, Boerma T. Health information systems: the foundations of public health. Bull World Health Organ. 2005 Aug;83(8):578–83.

3. World Health Organization. Everybody’s business: strengthening health systems to improve health outcomes : WHO’s frmaework for action. Geneva: World Health Organization; 2007.

4. Health Metrics Network, World Health Organization. Framework and standards for country health information systems. Geneva, Switzerland: WHO; 2008.

5. Nisingizwe MP, Iyer HS, Gashayija M, Hirschhorn LR, Amoroso C, Wilson R, et al. Toward utilization of data for program management and evaluation: quality assessment of five years of health management information system data in Rwanda. Glob Health Action [Internet]. 2014 Nov 19 [cited 2018 Aug 13];7. Available from: https://www.ncbi.nlm.nih.gov/pmc/articles/PMC4238898/

6. Amoakoh-Coleman M, Kayode GA, Brown-Davies C, Agyepong IA, Grobbee DE, Klipstein-Grobusch K, et al. Completeness and accuracy of data transfer of routine maternal health services data in the greater Accra region. BMC Res Notes [Internet]. 2015 Apr 1 [cited 2018 Aug 13];8. Available from: https://www.ncbi.nlm.nih.gov/pmc/articles/PMC4392754/

7. Roomaney RA, Pillay-van Wyk V, Awotiwon OF, Nicol E, Joubert JD, Bradshaw D, et al. Availability and quality of routine morbidity data: review of studies in South Africa. J Am Med Inform Assoc. 2017 Apr 1;24(e1):e194–206.

8. Mphatswe W, Mate K, Bennett B, Ngidi H, Reddy J, Barker P, et al. Improving public health information: a data quality intervention in KwaZulu-Natal, South Africa. Bull World Health Organ. 2012 Mar 1;90(3):176–82.

9. Joubert J, Rao C, Bradshaw D, Vos T, Lopez AD. Evaluating the Quality of National Mortality Statistics from Civil Registration in South Africa, 1997–2007. Leone T, editor. PLoS ONE. 2013 May 27;8:e64592.

10. O’Hagan R, Marx MA, Finnegan KE, Naphini P, Ng’ambi K, Laija K, et al. National Assessment of Data Quality and Associated Systems-Level Factors in Malawi. Glob Health Sci Pract. 2017 Sep 27;5(3):367–81.

11. Kihuba E, Gathara D, Mwinga S, Mulaku M, Kosgei R, Mogoa W, et al. Assessing the ability of health information systems in hospitals to support evidence-informed decisions in Kenya. Glob Health Action. 2014 Dec 1;7(1):24859.

12. de Souza DK, Yirenkyi E, Otchere J, Biritwum N-K, Ameme DK, Sackey S, et al. Assessing Lymphatic Filariasis Data Quality in Endemic Communities in Ghana, Using the Neglected Tropical Diseases Data Quality Assessment Tool for Preventive Chemotherapy. PLoS Negl Trop Dis [Internet]. 2016 Mar 30 [cited 2018 Aug 13];10(3). Available from: https://www.ncbi.nlm.nih.gov/pmc/articles/PMC4814091/

13. Chiba Y, Oguttu MA, Nakayama T. Quantitative and qualitative verification of data quality in the childbirth registers of two rural district hospitals in Western Kenya. Midwifery. 2012 Jun;28(3):329–39.

14. Sharma A, Rana SK, Prinja S, Kumar R. Quality of Health Management Information System for Maternal & Child Health Care in Haryana State, India. PLoS ONE [Internet]. 2016 [cited 2018 Oct 24];11(2). Available from: https://www.ncbi.nlm.nih.gov/pmc/articles/PMC4752326/

15. Hahn D, Wanjala P, Marx M. Where is information quality lost at clinical level? A mixed-method study on information systems and data quality in three urban Kenyan ANC clinics. Glob Health Action. 2013 Dec;6(1):21424.

16. Zulu Z, Kunene S, Mkhonta N, Owiti P, Sikhondze W, Mhlanga M, et al. Three parallel information systems for malaria elimination in Swaziland, 2010–2015: are the numbers the same? Public Health Action. 2018 Apr 25;8(Suppl 1):S13–7.

17. Lippeveld T. Routine Health Facility and Community Information Systems: Creating an Information Use Culture. Glob Health Sci Pract. 2017 Sep 27;5(3):338–40.

18. Biruk S, Yilma T, Andualem M, Tilahun B. Health Professionals’ readiness to implement electronic medical record system at three hospitals in Ethiopia: a cross sectional study. BMC Med Inform Decis Mak. 2014 Dec 12;14:115.

19. Shiferaw AM, Zegeye DT, Assefa S, Yenit MK. Routine health information system utilization and factors associated thereof among health workers at government health institutions in East Gojjam Zone, Northwest Ethiopia. BMC Med Inform Decis Mak [Internet]. 2017 Aug 7 [cited 2018 Oct 30];17. Available from: https://www.ncbi.nlm.nih.gov/pmc/articles/PMC5545835/

20. Nicol E, Bradshaw D, Uwimana-Nicol J, Dudley L. Perceptions about data-informed decisions: an assessment of information-use in high HIV-prevalence settings in South Africa. BMC Health Serv Res [Internet]. 2017 Nov [cited 2018 Aug 13];17(S2). Available from: https://bmchealthservres.biomedcentral.com/articles/10.1186/s12913-017-2641-1

21. Dagnew E, Woreta SA, Shiferaw AM. Routine health information utilization and associated factors among health care professionals working at public health institution in North Gondar, Northwest Ethiopia. BMC Health Serv Res [Internet]. 2018 Sep 4 [cited 2018 Oct 30];18. Available from: https://www.ncbi.nlm.nih.gov/pmc/articles/PMC6122568/

22. Mutale W, Chintu N, Amoroso C, Awoonor-Williams K, Phillips J, Baynes C, et al. Improving health information systems for decision making across five sub-Saharan African countries: Implementation strategies from the African Health Initiative. BMC Health Serv Res. 2013 May 31;13(2):S9.

23. Nutley T, Gnassou L, Traore M, Bosso AE, Mullen S. Moving data off the shelf and into action: an intervention to improve data-informed decision making in Côte d’Ivoire. Glob Health Action [Internet]. 2014 Oct 1 [cited 2018 Oct 30];7. Available from: https://www.ncbi.nlm.nih.gov/pmc/articles/PMC4185136/

24. Tuti T, Bitok M, Paton C, Makone B, Malla L, Muinga N, et al. Innovating to enhance clinical data management using non-commercial and open source solutions across a multi-center network supporting inpatient pediatric care and research in Kenya. J Am Med Inform Assoc JAMIA. 2016 Jan;23(1):184–92.

25. Wagenaar BH, Gimbel S, Hoek R, Pfeiffer J, Michel C, Manuel JL, et al. Effects of a health information system data quality intervention on concordance in Mozambique: time-series analyses from 2009–2012. Popul Health Metr [Internet]. 2015 Mar 26 [cited 2019 Jan 27];13. Available from: https://www.ncbi.nlm.nih.gov/pmc/articles/PMC4377037/

26. WHO | Data Quality Review (DQR) Toolkit [Internet]. WHO. [cited 2018 Oct 23]. Available from: http://www.who.int/healthinfo/tools_data_analysis/dqr_modules/en/

27. Routine Data Quality Assessment Tool - User Manual — MEASURE Evaluation [Internet]. [cited 2019 Jan 30]. Available from: https://www.measureevaluation.org/resources/publications/ms-17-117

28. Tricco AC, Lillie E, Zarin W, O’Brien KK, Colquhoun H, Levac D, et al. PRISMA Extension for Scoping Reviews (PRISMA-ScR): Checklist and Explanation. Ann Intern Med. 2018 Oct 2;169(7):467.
