# Supplementary material for: Improving quality and use of routine health information system data in low- and middle-income countries: A scoping review
Source: PLoS One. 2020 Oct 8;15(10):e0239683. doi: 10.1371/journal.pone.0239683 (PMC7544093; doi:10.1371/journal.pone.0239683)
Supplement: Attachment — Submitted filename: PONE-D-20-12315_reviewer (1).pdf [file pone.0239683.s005.pdf]

PLOS ONE

Improving quality and use of routine health information system data in low- and middle-income countries: A scoping review

--Manuscript Draft--

**Manuscript Number:**	PONE-D-20-12315
**Article Type:**	Research Article
**Full Title:**	Improving quality and use of routine health information system data in low- and middle-income countries: A scoping review
**Short Title:**	Improving quality and use of routine health information system data in low- and middle-income countries
**Corresponding Author:**	Sebelewengel Lemma<br>London School of Hygiene and Tropical Medicine<br>Addis Ababa, ETHIOPIA
**Keywords:**	Performance of Routine Information System Management (PRISM); data quality; data use; decision making; low- and middle-income countries
**Abstract:**	[b]Background:[/b] A routine health information system is one of the essential components of a health system. Interventions to improve routine data quality and use for decision-making in low- and middle-income countries differ in design, methods, and scope. There have been limited efforts to synthesise the knowledge across the currently available intervention studies. Thus, this scoping review synthesised published results from interventions that aimed at improving data quality and use in routine health information systems in low- and middle-income countries.<br>[b]Method:[/b] We included articles on intervention studies that aimed to improve data quality and use within routine health information systems in low- and middle-income countries, published in English from January 2008 to February 2020. We searched the literature in the databases Medline/PubMed, Web of Science, and Global Health. After a meticulous screening, we identified 20 articles on data quality and 16 on data use. We prepared and presented the results as a narrative. [b]Results:[/b] Most of the studies were from Sub-Saharan Africa and designed as case studies. Interventions enhancing the quality of data targeted health facilities with staff at the district level, and targeted district health managers for improved data use. Combinations of technology enhancement along with capacity building activities, and data quality assessment and feedback system were found useful in improving data quality. Interventions facilitating data availability combined with technology enhancement increased the use of data for planning. [b]Conclusion:[/b] The studies in this scoping review gave due emphasis to the use of a combination of interventions at a given time, addressing both the behavioural and technical factors of routine health information system performance improved data quality and use. Interventions addressing organisational factors were not existing, but these factors were reported to pose challenges to the implementation and performance of reported interventions. This work was supported by the Bill & Melinda Gates Foundation, Grant Number OPP1187448
**Order of Authors:**	Seblewengel Lemma Abreham
	Annika Janson
	Lars Ake Persson
	Deepthi Wickremasinghe
	Carina Kallestal
**Additional Information:**	
**Question**	**Response**
**Financial Disclosure**<br><br>Enter a financial disclosure statement that describes the sources of funding for the	The authors received no specific funding for this work.

Powered by Editorial Manager® and ProduXion Manager® from Aries Systems Corporation

work included in this submission. Review the submission guidelines for detailed requirements. View published research articles from *PLOS ONE* for specific examples.<br><br>This statement is required for submission and **will appear in the published article** if the submission is accepted. Please make sure it is accurate.<br><br>**Unfunded studies**<br>Enter: *The author(s) received no specific funding for this work.*<br><br>**Funded studies**<br>Enter a statement with the following details:<br>• Initials of the authors who received each award<br>• Grant numbers awarded to each author<br>• The full name of each funder<br>• URL of each funder website<br>• Did the sponsors or funders play any role in the study design, data collection and analysis, decision to publish, or preparation of the manuscript?<br>• **NO** - Include this sentence at the end of your statement: *The funders had no role in study design, data collection and analysis, decision to publish, or preparation of the manuscript.*<br>• **YES** - Specify the role(s) played.<br><br>* typeset	
**Competing Interests**<br><br>Use the instructions below to enter a competing interest statement for this submission. On behalf of all authors, disclose any competing interests that could be perceived to bias this work—acknowledging all financial support and any other relevant financial or non-financial competing interests.<br><br>This statement **will appear in the published article** if the submission is accepted. Please make sure it is accurate. View published research articles from *PLOS ONE* for specific examples.	The authors have declared that no competing interests exist.

Powered by Editorial Manager® and ProduXion Manager® from Aries Systems Corporation

**NO authors have competing interests**<br><br>Enter: *The authors have declared that no competing interests exist.*<br><br>**Authors with competing interests**<br><br>Enter competing interest details beginning with this statement:<br><br>*I have read the journal's policy and the authors of this manuscript have the following competing interests: [insert competing interests here]*<br><br>* typeset	
**Ethics Statement**<br><br>Enter an ethics statement for this submission. This statement is required if the study involved:<br><br>• Human participants<br>• Human specimens or tissue<br>• Vertebrate animals or cephalopods<br>• Vertebrate embryos or tissues<br>• Field research<br><br>Write "N/A" if the submission does not require an ethics statement.<br><br>General guidance is provided below. Consult the submission guidelines for detailed instructions. **Make sure that all information entered here is included in the Methods section of the manuscript.**	N/A

Powered by Editorial Manager® and ProduXion Manager® from Aries Systems Corporation

**Format for specific study types**<br><br>**Human Subject Research (involving human participants and/or tissue)**<br>• Give the name of the institutional review board or ethics committee that approved the study<br>• Include the approval number and/or a statement indicating approval of this research<br>• Indicate the form of consent obtained (written/oral) or the reason that consent was not obtained (e.g. the data were analyzed anonymously)<br><br>**Animal Research (involving vertebrate animals, embryos or tissues)**<br>• Provide the name of the Institutional Animal Care and Use Committee (IACUC) or other relevant ethics board that reviewed the study protocol, and indicate whether they approved this research or granted a formal waiver of ethical approval<br>• Include an approval number if one was obtained<br>• If the study involved *non-human primates*, add *additional details* about animal welfare and steps taken to ameliorate suffering<br>• If anesthesia, euthanasia, or any kind of animal sacrifice is part of the study, include briefly which substances and/or methods were applied<br><br>**Field Research**<br><br>Include the following details if this study involves the collection of plant, animal, or other materials from a natural setting:<br>• Field permit number<br>• Name of the institution or relevant body that granted permission	
**Data Availability**<br><br>Authors are required to make all data underlying the findings described fully available, without restriction, and from the time of publication. PLOS allows rare exceptions to address legal and ethical concerns. See the PLOS Data Policy and FAQ for detailed information.	Yes - all data are fully available without restriction

A Data Availability Statement describing where the data can be found is required at submission. Your answers to this question constitute the Data Availability Statement and **will be published in the article**, if accepted.<br><br>**Important:** Stating 'data available on request from the author' is not sufficient. If your data are only available upon request, select 'No' for the first question and explain your exceptional situation in the text box.<br><br>Do the authors confirm that all data underlying the findings described in their manuscript are fully available without restriction?	
**Describe where the data may be found in full sentences. If you are copying our sample text, replace any instances of XXX with the appropriate details.**<br><br>• If the data are **held or will be held in a public repository**, include URLs, accession numbers or DOIs. If this information will only be available after acceptance, indicate this by ticking the box below. For example: *All XXX files are available from the XXX database (accession number(s) XXX, XXX.*).<br>• If the data are all contained **within the manuscript and/or Supporting Information files**, enter the following: *All relevant data are within the manuscript and its Supporting Information files.*<br>• If neither of these applies but you are able to provide **details of access elsewhere**, with or without limitations, please do so. For example:<br><br>*Data cannot be shared publicly because of [XXX]. Data are available from the XXX Institutional Data Access / Ethics Committee (contact via XXX) for researchers who meet the criteria for access to confidential data.*<br><br>*The data underlying the results presented in the study are available from (include the name of the third party*	All relevant data are within its supporting information files.

and contact information or URL).<br>• This text is appropriate if the data are owned by a third party and authors do not have permission to share the data.<br><br>* typeset	
Additional data availability information:	

Click here to access/download;Manuscript;PublicationFiles-manuscript_scoping_final_15_04_2020.docx

Improving quality and use of routine health information system data in low- and middle-income countries: A scoping review

Seblewengel Lemma[1], Annika Janson[1,#a], Lars-Åke Persson[1], Deepthi Wickremasinghe[2+], Carina Källestål[1,#b]

[1] Department of Disease control, London School of Hygiene and Tropical Medicine, based at the Ethiopian Public Health Institute, Addis Ababa, Ethiopia

[2] Department of Disease Control, London School of Hygiene & Tropical Medicine, London, UK

[#a] Department of Women's and Children's Health, Karolinska Institutet, Stockholm, Sweden

[#b] Department of Women's and Children's Health, Uppsala University, Uppsala, Sweden

[+] Deceased

*Corresponding author

Email: lemmaseblewengel@gmail.com; Seblewengel.abreham@lshtm.ac.uk

Authors' contributions:

SL: Developed the review protocol, served as second reader during screening of study articles, interpreted the findings, and wrote all versions of the manuscript. AJ: Served as first reader during the screening of study articles, and contributed to revisions of the manuscript. LP and DW: Contributed to the research protocol and revisions of the manuscript. CK: Contributed in the inception of the research idea, protocol development, served as third reviewer during the screening of study articles, and contributed to revisions of the manuscript. SL, AJ, LP, and CK approved the final version of the manuscript.

Abstract

Background: A routine health information system is one of the essential components of a health system. Interventions to improve routine data quality and use for decision-making in low- and middle-income countries differ in design, methods, and scope. There have been limited efforts to synthesise the knowledge across the currently available intervention studies. Thus, this scoping review synthesised published results from interventions that aimed at improving data quality and use in routine health information systems in low- and middle-income countries.

Method: We included articles on intervention studies that aimed to improve data quality and use within routine health information systems in low- and middle-income countries, published in English from January 2008 to February 2020. We searched the literature in the databases Medline/PubMed, Web of Science, and Global Health. After a meticulous screening, we identified 20 articles on data quality and 16 on data use. We prepared and presented the results as a narrative.

Results: Most of the studies were from Sub-Saharan Africa and designed as case studies. Interventions enhancing the quality of data targeted health facilities with staff at the district level, and targeted district health managers for improved data use. Combinations of technology enhancement along with capacity building activities, and data quality assessment and feedback system were found useful in improving data quality. Interventions facilitating data availability combined with technology enhancement increased the use of data for planning.

Conclusion: The studies in this scoping review gave due emphasis to the use of a combination of interventions at a given time, addressing both the behavioural and technical

factors of routine health information system performance improved data quality and use. Interventions addressing organisational factors were not existing, but these factors were reported to pose challenges to the implementation and performance of reported interventions.

Keywords: Performance of Routine Information System Management (PRISM), data quality, data use, decision making, low- and middle-income countries

Background

A health information system is one of the six building blocks of a health system and is designed to meet information needs within the health system. It generates routine health information that is vital for planning, monitoring, and evaluating public health programs and interventions (1–3). Decisions are made continuously at all levels of the health system. Information is generated that influences decisions from patient care to policy formation and implementation, thereby influencing health in the communities served by the health system (1). The health information system generates information mainly from the routine health information system, which is composed of health service-based data as well as population-based data from surveys, census, and vital event registrations (4). The health information system performance is expressed as the quality of these data and their use for decision-making (1).

Thus, the quality of routine data is vital for the health system to function well and for policymakers to be able to evaluate the effects of health system efforts to improve the health of the population (2). The quality of the routine health information system data has been enhanced across the globe (5,6). However, health systems in low- and middle-income countries are still suffering from a suboptimal quality and inadequate use of data generated by their routine health information systems (7–9). The data quality issues are often expressed as incomplete registers (10,11), lack of consistency between registers and reports (12–15), and a low level of data accuracy (9). Discrepancies between results from data generated in the routine health information system and population-based surveys are common (11).

Despite the increasing availability of health information, the use of such information for decision-making is still deficient in many low- and middle-income countries (16). Studies from these settings show limited or inadequate use of data, especially of routinely generated data (17–20). Studies at health facilities or based on interviews with health workers have frequently reported low use of such data for planning, despite them being engaged in the collection, aggregation, and generation of data reports to the next level of the health system (18,20,21). A lack of trust in the quality of data may partly explain the limited use of data (17,20).

The potential factors that affect routine health information system performance are classified as technical, behavioural, and organizational factors according to the performance of routine health information system (PRISM) framework (21). Given the vital role of the routine health information system, there have been several interventions aiming to improve the quality and use of data targeting these potential factors (8,22–26). The design, methods, and scope of these interventions vary, making it difficult to conclude which interventions could be successful in what contexts.

The London School of Hygiene and Tropical Medicine implemented an intervention project called Operational Research and Coaching for Analysts (ORCA) in collaboration with the Ethiopian Public Health Institute and the Ethiopian Federal Ministry of Health. This project aimed to contribute to the improvement of data quality and use in the Ethiopian routine health information system. This scoping review was part of these efforts and focused on the evaluation and synthesis of published results from interventions that aimed at improving data quality and use in routine health information systems in low- and middle-income countries. It answers the question: which interventions have successfully enhanced the

quality and use of routine health information system data in low- and middle-income countries, and what contextual factors have influenced that outcome?

Methods

Protocol and registration

We developed a protocol and before starting the work however we were not able to register at on PROSPERO since it has stopped registering systematic review at the time of request. S3 file.

Study eligibility criteria

Peer-reviewed scientific journal papers were included in this review if the studies described or assessed interventions aimed at improving data quality or data use in the routine health information system. The studies should have been conducted in the health system in low- and middle-income countries and published in English in the period from January 2008 to February 2020. See Table 1 for inclusion and exclusion criteria.

We also included studies with any epidemiological design aiming at evaluating or describing a given intervention on the subject of interest, as well as qualitative studies of eva[illegible]tion nature.

Data quality is commonly defined based on its attributes or dimensions, and these dimensions vary with the different tools used. For instance, the WHO data quality review tool represents routine data quality as data completeness, timeliness, consistency, and accuracy (27). The routine data quality assessment tool prepared by the MEASURE evaluation group the data quality into a primary dimension that consists of accuracy and reliability and the sub-dimensions precision, completeness, timeliness, integrity, and

confidentiality (28). Data use was defined as the use of routine health information system data for decision making at any level of the health system.

Table 1: Summary of inclusion and exclusion criteria.

Inclusion criteria	Exclusion criteria
• Studies published in peer-reviewed scientific journals of any design meant to evaluate or describe interventions used to improve data quality or use of data from routine health information system	• Studies not focusing on routine health information system<br>• Grey literature<br>• Systematic reviews
• Studies published in English	• Language other than English
• Studies published from January 2008 to February 2020	• Studies published before January 2008
• Studies conducted in low- and middle-income countries according to the Development Assistance Committee (DAC)* list, 2018	• Studies conducted in high-income countries

*DAC: Development assistance committee

Information source, search strategy, and selection

To identify potentially relevant articles, we searched the following bibliographic databases from 2008 to 2018, with an additional search until February 2020: EMBASE, MEDLINE/PubMed, Web of Science, and Global Health. Combinations of search terms were prepared based on the objective of the scoping review (SL) and further reviewed by an experienced information scientist (DW) to increase the certainty that the combination of the search terms answered our objectives. We reviewed reference lists of published studies to check for saturation. The combination of search terms used is presented in S1A and S1B boxes.

We exported the retrieved studies to the Zotero reference manager (Corporation for Digital Scholarship, State, Country) and checked for duplicates. The first two reviewers (SL and AJ) screened titles and abstracts of the articles based on the eligibility criteria before reading

the full articles in the Zotero reference manager. A third reviewer (CK) reviewed both titles and abstracts of the articles in case of disagreement between the first two reviewers. The decision made by the third reviewer was the final for inclusion or exclusion of the disputed studies. The first two reviewers evaluated the selected full articles, and the third reviewer resolved any disagreements between the two reviewers.

Data charting

We exported a list of the included studies into an Excel sheet. An Excel data abstraction tool was prepared, tested, and modified accordingly. The first two reviewers independently charted and compared the extracted data for any significant variation. The third reviewer further reviewed articles with a significant difference for a final decision. (S1C Box.)

Synthesis

The presentation of the results followed the checklist for reporting of a scoping review "Preferred Reporting Items for Systematic Reviews and Meta-Analysis: extension for Scoping Reviews (PRISMA-ScR)" (28). S4file. We described the search approach, and prepared tables summarising the characteristics of the included studies and their results. The analysis synthesised the type of interventions applied to improve data quality and use, as well as the effect of the reported interventions. The study also narrated potential opportunities and challenges of the reported interventions that may have influenced the outcome in low- and middle-income countries.

Results

Selection of sources of evidence

A total of 12 studies on the quality of data and 13 on data use were identified. Two studies were captured in both (data quality and data use) search processes. Three studies identified by the data use search process also dealt with data quality and were added to the data quality literature that finally included a total of 15 scientific papers. From the additional search for studies published January 2019- February 2020, we found one study that addressed data use alone, and five studies on data quality of which, two were both on data quality and use. In total, we evaluated 20 and 16 studies on interventions on quality and use of data, respectively. The following flow diagram (Figure 1 and Figure 2) depicts the process of selection of literature and the search criteria.

Figure 1: The process and results of the literature search for data quality

Figure 2: The process and results of the literature search for data use

Characteristics of the literature

Out of the total of 20 studies on data quality, 14 were conducted in Africa, mostly in Sub-Saharan Africa (29–31,25,32–41), three in Asia (India, Pakistan, Siri Lanka) (42–44), and the remaining three in South America (Brazil, Mexico &Peru and Haiti) (45–47). Out of the 17 articles included in the review of data use, ten were from Africa (36,37,39,48–54), mostly the Sub-Saharan region, six were from Asia (Philippines, Bangladesh, Siri Lanka, India, and Iran) (26,43,44,55–57), and one from the Caribbean (Haiti) (58).

Most studies used a combination of interventions, such as the introduction of technology with training and supportive supervision. In general, the interventions on data quality fell into three major areas. The first and most common intervention on data quality was the use

of technology, such as personal digital assistants (PDAs) for data entry, electronic form of data collection tools, such as electronic medical record (EMR), and electronic health management information system (EHMIS) to improve the data capturing at the health facility level. The second group of interventions was capacity building activities for personnel engaged in data collection, processing, and reporting at the health facility up to district level. The training was mainly on self-assessment and data quality management activities and how to use a framework for continuous improvements, such as the modified Plan-Do-Study-Act framework to systematically identify and act on data quality issues. The third group of interventions used evaluation tools to improve self-assessment and feedback systems in the routine health information system. These interventions encouraged the regular provision of feedback based on a systematic assessment of the data quality in the routine health information system. Table 2 summarises the data quality interventions.

Table 2. Interventions tested or adopted on specific health system populations aimed to improve data quality.

Author	Population	Interventions
1. Use of technology		
Ndira SP., 2008 (36)	Health system	Electronic health management information system compared to paper-based
Maokola W., 2011(29)	Data collectors at health centres	Personal digital assistants
Monda J., 2012 (31)	Clinic health record system	Data integrity module: Plugin technology for auto-identification and correction of data capturing errors
Mutale W., 2013 (25)	Health system	Simplified information capturing system
Gimbel S., 2017(34)	Health facilities and managers at the district level	Enhanced electronic medical record
Kaushik A., 2015 (43)	Secondary & tertiary care hospitals, primary care health centres and medical colleges	Enterprise architecture for e-health: web-based health management information system solution

Dombrowski JG., 2015 (45)	Routine health information system	Vertical health information system; live birth information system
Tuti T., 2016 (33)	Hospitals and clinicians treating children	Electronic tools. Research electronic data capture
Ismail S., 2017 (42)	Maternal and child health clinics	Standard-compliant data access model for maternal and child health data
Trumbo SP., 2018 (47)	Health facilities	Electronic immunization registers with multiple data-capturing systems
Rado, R., 2018 (35)	Health care structures in districts	Reinforced integrated disease surveillance response: Use of SMS for data transfer
Lazzerini, M., 2019 (44)	Hospitals	Development of an individual patient prospective database
2. Capacity-building activities		
Braa J., 2012 (30)	District health management team	Data use workshops for users
Mutali W., 2013(25)	Health facility, district and province	Training of personnel managing data on data quality
Mpofu M., 2014 (32)	District monitoring and evaluation officers	Training and deploying monitoring and evaluation officers to manage data at the district
Wagenaar BH., 2017 (37)	Project principal investigators, implementers and Ministry of Health	Training of sub-national level managers on data analysis and output presentation approach
Gimbel S., 2017(34)	Health facilities and managers at the district level	Continuous on-site mentorship
Njuguna, C., 2019 (40)	Health facilities	Development of customized guidelines for intergraded disease surveillance and response; training of focal person;
3. Data quality assessments and feedback mechanisms		
Mutale W., 2013 (25)	Health system: Districts and province, health centres, community health information system, dispensary and hospital	Routine provision of feedback on selected data quality issues; annual data quality assessment and feedback, community-level data assessment quarterly using Lots quality assurance. Quarterly data quality audit and automated data quality report based on logic error. Regular (monthly) review of reports and planned meetings between data generators and supervisors; district performance review and enhancement meetings.
Tuti T., 2016 (33)	Hospitals and clinicians treating children	Routine provision of feedback on data and mentorship; regular meetings three times a year
Puttkamer N., 2017 (46)	Site managers, clinicians, and data officers from hospitals and clinics using iSante	Automated data queries for extensive scale-site electronic medical record system (two strategies to establish priorities for data quality assessment)

Wagenaar B.H., 2017(37)	Project principal investigators, implementers and Ministry of Health	Modified Plan-Do-Study-Act framework: facility-based data quality assessment, data review, and feedback meetings, data-driven action plan and follow-up of the plan
Gimbel S., 2017(34)	Health facilities and managers at the district level	Developed data quality assessment for community health information systems, conducted quarterly data quality assessments, monthly data review meetings, and targeted supportive supervision
Njuguna, C., 2019 (40)	Health facilities	Monitoring, supervision and data quality assessment
O'Connor, EC., 2019 (39)	Community health workers and community leaders	Participatory community-based health information system
Yourkavitch, J., 2019 (41)	Community health workers	Data quality assessments to stimulate improvements to health management information systems

* iSante: a multi-site electronic medical record implemented by the Haitian Ministry of Health.

Most of the studies on data use included combined interventions. These interventions also fell into three groups depending on the data use attributes the authors tried to improve or influence. The first group of interventions was involved in the use of decision-making facilitation tools or models. These tools or models should help decision-making by availing the necessary information in a logical and meaningful manner. The second group of interventions dealt with the use of technology to improve data quality and data use. These interventions also aimed to improve the availability of data for decision making. The third group of interventions was capacity building interventions, and only three articles fell under this category. Table 3 shows the details.

Table 3. Interventions tested or adopted on specific health system populations aimed to improve data use

Author	Population	Interventions
1. Decision-making facilitation tools or models		
La Vincente S., 2013 (55)	Rural province and urbanized city	Investment case approach: a decision support model that estimate the cost and impact of alternative interventions

Nutlay T., 2013 (50)	Health facilities in districts	District Health Profile: an excel based system linked with routine data
Rajan D., 2014 (52)	Health System	Resource planning model based on the WHO integrated health care technology package
Kaushik A., 2015 (43)	Secondary and tertiary care hospitals, primary care health centres and medical colleges	Enterprise architecture for eHealth: Web-based health management information system solution that improves the availability of data for decision making
O'Connor, EC., 2019 (39)	Community health workers and community leaders	Participatory community-based health information system
2. Use of technology to improve data quality and use		
Gamur G., 2008 (48)	Primary care clinics	Feedback and Analytic Comparison tool: a form of a health information system at primary health care setting designed to facilitate data use for decision making
Ndira SP., 2008 (36)	Health system	Use of electronic health management information system to facilitate data quality and use
Matheson, AL., 2012 (58)	Hospitals where HIV care was provided	Use of electronic medical record system to facilitate data use
Hosseini M., 2014 (56)	Immunization records	Developing an immunization information system using service-oriented architecture and health level 7 to improve interoperability
Landis-Lewis Z., 2015 (53)	Anti-retroviral clinics and health care providers	Use of electronic medical record in anti-retroviral treatment clinics to improve the use of data for feedback
Mutale W., 2013 (25)	Clinics	Use of electronic data capture system to improve the quality of data improves data use at the health facility level
Mutale W., 2013 (25)	Community health information system workers at the community, dispensary, health centre and hospitals	Linking community-level health information data with routine health information system at facility and district
Nakibuuka, J., 2019 (38)	Health facilities	An Unstructured Supplementary Service Data-based health data reporting
Lazzerini, M., 2019 (44)	Hospitals	Development of a prospective Individual patient prospective database
3. Capacity building to foster data use		
Braa J., 2012 (30)	District Health Management team	Quarterly data use workshops for district health management team
Wagenaar BH., 2017 (37)	Project principal investigators, implementers, and Ministry of Health	Modified Plan-Do-Study-Act framework. Training of sub-national managers on data analysis and output presentation approach

Uneke JC., 2019 (54)	Policymakers	Policy information platform to improve access to information and thereby enhance the use of data for decision making and policy formation

Design and target population

A good number of the studies lacked a clear description of the study methodology, i.e., their design and other methodological information. Four of the articles that dealt with data quality were case studies, which described an intervention, its implementation, and the observed changes or improvement in data quality (25,31,33,46). Four reports had used a mixed-methods approach, where the authors had combined designs, including qualitative assessments (34,35,41,47). Six studies claimed to have an evaluation approach (30,32,35,38,39,42) but without any well-articulated evaluation design, and two of the evaluation studies had employed a qualitative approach (30,32). Two papers reported cross-sectional studies (29,45). A majority of the articles on the use of data were case studies (25,43,48,52,55,56,58), two had used a mixed-methods approach (36,54), three were qualitative studies (37,50,53), and two claimed evaluation designs that were not delin[illegible]ed (30,36).

The target population for the data quality interventions was mainly health facilities at the district level and or their staff, including health care providers and data generators (29,31,33,35,38–42,44). Other studies targeted both the health facilities at the district level and the district management team (25,34,46,47), and two reports solely focused on the district management team (30,32). The target population for data use interventions was similar to the data quality intervention studies. They focused on health facilities and their staff (48,50,53,57,58), or the health system in general, including its health management information system (36,55,56), or the management team, primarily at the district level

(30,37,54), or a combination of the health facility and the district management team (25). One study considered community health workers and the community health information system (25). (S2A and S2B Tables.)

The outcome of the interventions as reported by the studies

Most studies used combinations of interventions. Fourteen of the 20 studies on data quality reported changes in data quality after the interventions (25,29,30,32–34,36–40,44,45,47), and 11 of the 14 studies that reported change showed improvement in data quality (30,32–34,36–40,44,47). For instance, the study by Mopuf and colleagues from Botswana, reported improved routine data quality and reporting after training and deploying monitoring and evaluation officers at the district health office level (32). Gimbel and colleagues (34) appreciated the use of combined interventions. Such examples were annual data quality assessments, provision of feedback to all districts using a summary data quality ranking tool, targeted supportive supervision for sites with weaker performance, in addition to monitoring and evaluation training. The latter study reported improved data availability from 84% to 99%, and a change in data consistency from 54% to 87% in Mozambique, one of their target countries (34). Two of the studies reported a negative result (a decline in data quality performance) despite the interventions (29,45). A study in Tanzania introduced the use of personal data assistance and trained data collectors at the health centre level, combined with monthly supervision and discussion on monthly generated reports without any positive effect on register completeness (29). The second negative study established a live birth information system and compared its performance with an already available civil registry in Brazil (45). The live birth information system performed poorly in coverage and completeness compared to the existing civil registry. The rest of the studies described the

process and implementation of interventions that were meant to improve data quality without measuring the effect (31,41–43,46). (S2A Table.)

The studies on data use also tested combinations of interventions. Eleven studies reported improvement in data use (25,30,37,39,43,44,48,50,52,54,57). The study by Braa and colleagues in Zanzibar tested quarterly five-days use-of-data workshops for district health management teams included a peer review of performance on common data quality issues, after each team presenting their data. This study reported the adoption of simplified data capturing tools, increased use of data quality checks at facility levels and in districts, and improved alignment between plans, targets, and indicators (30). Nutley and colleagues also tested a decision support system labelled the district health profile in Kenya. This system was an Excel-based system with a set of standard questions on data quality. The authors reported improved data analysis, review, interpretation, and sharing of data, implying improved data use for decision making (50). Wagenaar and colleagues also reported that most respondents reached the consensus that data were correct after implementing a modified Plan-Do-Study-Act framework in Zambia, Mozambique, and Rwanda (37). This framework had steps such as identification of data quality problems, implementation of facility-based data quality assessment, training, feedback, data-driven action plan, supervision, and follow-up for action (37). The rest of the studies reported a process of adoption of use-of-data interventions such as electronic medical record systems and their usefulness and acceptance by the targeted groups without assessing their effect on data use (55,56,58). (S2B Table.)

Reported barriers and positive attributes affecting interventions

A limited number of studies mentioned barriers or positive factors that could have influenced the interventions. No study tested these factors quantitatively in a statistical

model. Besides, some of these factors reported were specific to each type of intervention assessed in the respective studies. We have summarized factors, which were crosscutting across the included papers. We broadly classified these factors as related staff, resources, or infrastructure. Factors related to staff were lack of knowledge, skills, or training on a specific program or intervention, and inability to carry out the needed activities correctly or according to a set guideline (25,35,42,45). Some studies mentioned the lack of commitment or motivation to carry out a task or to adopt a new technology, which could affect either the implementation, the outcome, or both (25,36,45). One study mentioned the lack of technical personnel as a barrier to reaching the intended result of the intervention (47). Issues related to leadership, such as, variation in the leadership quality and motivation of supervisors were reported as barrier (25). At the same time, the presence of regular feedback facilitated a positive outcome of the intervention (35). Lack of guidelines or protocols in the health system to guide the interventions (35), lack of resources in general, interruption of funding (47), and lack of technological infrastructures such as computers and poor network connectivity were reported as barriers (38,42).

Similarly, some studies that dealt with the use of data reported on the barriers or favourable factors. Broadly, the elements summarised as issues related to the data quality, users, and resources. Poor data quality (48) and a limited availability of data (53,55) were barriers to the implementation of data use interventions. Limited user acceptance of the intervention (53), limited capacity of users to access and use interventions (54), and users having little value for data or trust on the quality of data were also barriers (37,50). A persistent culture of non-use of data (37) was a barrier to the implementation as well as the outcome of these interventions. Some studies also reported resource constraints such as access to computers and internet connectivity as potential barriers to the success of interventions (50,58).

Discussion

In this scoping review, we identified 20 studies on data quality and 16 on data use. Most of these studies were from Sub-Saharan Africa, and most researchers had employed a case study approach. The main target groups for the interventions were district-level health facilities with staff or the public health system as such. Studies that dealt with the use of data for health planning also targeted district health managers. Interventions were frequently combined so that different aspects or attributes of data quality or use were emphasised. Most of these combined interventions reported good progress and improved data quality and use in their respective target populations.

Data quality interventions that combined different capacity building activities, such as training and onsite mentoring, were reportedly effective. The combinations of capacity building activities with enhanced technical tools and data quality assessment combined with feedback systems were also useful. Two studies that did not use a combination of interventions reported persistent poor data quality in the routine health information system (29,45). Both studies focused only on technology enhancement, such as the use of electronic data capture, and the reports commented that technology enhancement alone might not bring the intended change. The latter study recommended routine investment in capacity building and regular data quality assessments.

Similarly, the use-of-data studies also stressed the importance of a combination of interventions. Those interventions facilitating data availability using standard tools combined with technology enhancement, such as the use of electronic data capture systems, were found useful in fostering data use. The technology enhancement

interventions that aimed to improve data quality along with capacity building activities also reported positive changes in the use of data.

In light of the Performance of Routine Information System Management (PRISM) framework (21), the interventions in this scoping review targeted technical factors, which mainly dealt with the use of technology, and approaches that simplified activities in the routine health information system. The studies addressed factors, such as skills in performing data quality checks, problem-solving ability, and competence for the tasks in the health information system. Organizational factors were more rarely in focus, except for training and supervision. Overall, technical, organizational, and behavioural factors were partly addressed by the interventions. The relevance of untouched factors was undeniable, and they partially appear as listed barriers to the performance of the interventions, and the implementation process by the studies. Some of these untouched factors were related to routine health information system governance or leadership, such as lack of funding, weak demand to use data, and lack of motivation concerning data quality and use.

The reviewed interventions did not involve higher-level managers and experts in the health system, as recommended in the PRISM framework. Although data are generated at lower levels of the health system, the contribution of experts and managers at all levels of the health system is crucial to realize better data quality and ensure continuous use of data at all levels. Managers and experts at higher levels in the health system are sources for many of the organizational factors, such as governance, finance, and promotion of information use that influence the performance of the routine health information system.

Data quality and data use interventions in high-income countries heavily relied on the use of technology. Examples are the use of electronic medical or health record systems to improve

data quality (59–63) and approaches to enhance the interoperability of such data sources to enhance the availability of data and use (60,62,64,65). At the same time, these interventions were target-specific compared to the interventions in low- and middle-income countries, where targets were broader. Further, interventions in high-income countries primarily target health facilities. In contrast, studies in low- and middle-income countries addressed health service managers at the district level. Such variations could be explained by the difference in the relative importance of factors presented in the PRISM framework in these two different settings. Otherwise, both settings took advantage of the ever-changing technology advancement to improve the performance of their routine health information system.

This scoping review has some limitations. Most of the scientific reports reviewed were case studies and lacked methodological rigour in evaluating a given intervention. It was not possible to assess effect sizes when studies reported improvement in data quality or use. Changes in the outcome were reported as a change in percentage, or the authors used qualitative statements to show the presence of increment or improvement in the intended outcome, without providing test statistics. Thus, the results on the effectiveness of the interventions reported by the individual studies must be interpreted with care. Furthermore, since this review included only published articles limited to low-and middle-income countries, it may not be representative of all available literature in the field and missed the experience from high-income countries.

Conclusion

The interventions reported in the reviewed studies targeted multiple dimensions of data quality and use. They called for combinations of interventions to enhance the performance of the routine health information system. There were positive effects when addressing both behavioural and technical factors in the routine health information system at the district health system level. There were few initiatives to target organizational factors that still pose a challenge to the performance of the system. Future routine health information system interventions should include organizational factors to influence the overall culture of data quality and use and also involve higher-level staff. Furthermore, intervention studies should employ an appropriate evaluation methodology that allows measures of the effect of an intervention.

Acknowledgements

We are greatly indebted to our friend and colleague, Ms. Deepthi Wickremasinghe, who co-authored this article but sadly passed away before it was published. We are also thankful for the support from the library at London School of Hygiene and Tropical Medicine that availed some of the literature.

References

1. Strengthening Health Information Systems in Low- and Middle-Income Countries—A Model to Frame What We Know and What We Need to Learn — MEASURE Evaluation [Internet]. [cited 2019 Jan 24]. Available from: https://www.measureevaluation.org/resources/publications/tr-17-156

2. AbouZahr C, Boerma T. Health information systems: the foundations of public health. Bull World Health Organ. 2005 Aug;83(8):578–83.

3. World Health Organization. Everybody's business: strengthening health systems to improve health outcomes : WHO's frmaework for action. Geneva: World Health Organization; 2007.

4. Health Metrics Network, World Health Organization. Framework and standards for country health information systems. Geneva, Switzerland: WHO; 2008.

5. Nisingizwe MP, Iyer HS, Gashayija M, Hirschhorn LR, Amoroso C, Wilson R, et al. Toward utilization of data for program management and evaluation: quality assessment of five years of health management information system data in Rwanda. Glob Health Action [Internet]. 2014 Nov 19 [cited 2018 Aug 13];7. Available from: https://www.ncbi.nlm.nih.gov/pmc/articles/PMC4238898/

6. Amoakoh-Coleman M, Kayode GA, Brown-Davies C, Agyepong IA, Grobbee DE, Klipstein-Grobusch K, et al. Completeness and accuracy of data transfer of routine maternal health services data in the greater Accra region. BMC Res Notes [Internet]. 2015 Apr 1 [cited 2018 Aug 13];8. Available from: https://www.ncbi.nlm.nih.gov/pmc/articles/PMC4392754/

7. Roomaney RA, Pillay-van Wyk V, Awotiwon OF, Nicol E, Joubert JD, Bradshaw D, et al. Availability and quality of routine morbidity data: review of studies in South Africa. J Am Med Inform Assoc. 2017 Apr 1;24(e1):e194–206.

8. Mphatswe W, Mate K, Bennett B, Ngidi H, Reddy J, Barker P, et al. Improving public health information: a data quality intervention in KwaZulu-Natal, South Africa. Bull World Health Organ. 2012 Mar 1;90(3):176–82.

9. de Souza DK, Yirenkyi E, Otchere J, Biritwum N-K, Ameme DK, Sackey S, et al. Assessing Lymphatic Filariasis Data Quality in Endemic Communities in Ghana, Using the Neglected Tropical Diseases Data Quality Assessment Tool for Preventive Chemotherapy. PLoS Negl Trop Dis [Internet]. 2016 Mar 30 [cited 2018 Aug 13];10(3). Available from: https://www.ncbi.nlm.nih.gov/pmc/articles/PMC4814091/

10. Chiba Y, Oguttu MA, Nakayama T. Quantitative and qualitative verification of data quality in the childbirth registers of two rural district hospitals in Western Kenya. Midwifery. 2012 Jun;28(3):329–39.

11. Sharma A, Rana SK, Prinja S, Kumar R. Quality of Health Management Information System for Maternal & Child Health Care in Haryana State, India. PLoS ONE [Internet]. 2016 [cited 2018 Oct 24];11(2). Available from: https://www.ncbi.nlm.nih.gov/pmc/articles/PMC4752326/

12. O'Hagan R, Marx MA, Finnegan KE, Naphini P, Ng'ambi K, Laija K, et al. National Assessment of Data Quality and Associated Systems-Level Factors in Malawi. Glob Health Sci Pract. 2017 Sep 27;5(3):367–81.

13. Kihuba E, Gathara D, Mwinga S, Mulaku M, Kosgei R, Mogoa W, et al. Assessing the ability of health information systems in hospitals to support evidence-informed decisions in Kenya. Glob Health Action. 2014 Dec 1;7(1):24859.

14. Hahn D, Wanjala P, Marx M. Where is information quality lost at clinical level? A mixed-method study on information systems and data quality in three urban Kenyan ANC clinics. Glob Health Action. 2013 Dec;6(1):21424.

15. Zulu Z, Kunene S, Mkhonta N, Owiti P, Sikhondze W, Mhlanga M, et al. Three parallel information systems for malaria elimination in Swaziland, 2010–2015: are the numbers the same? Public Health Action. 2018 Apr 25;8(Suppl 1):S13–7.

16. Lippeveld T. Routine Health Facility and Community Information Systems: Creating an Information Use Culture. Glob Health Sci Pract. 2017 Sep 27;5(3):338–40.

17. Biruk S, Yilma T, Andualem M, Tilahun B. Health Professionals' readiness to implement electronic medical record system at three hospitals in Ethiopia: a cross sectional study. BMC Med Inform Decis Mak. 2014 Dec 12;14:115.

18. Shiferaw AM, Zegeye DT, Assefa S, Yenit MK. Routine health information system utilization and factors associated thereof among health workers at government health institutions in East Gojjam Zone, Northwest Ethiopia. BMC Med Inform Decis Mak [Internet]. 2017 Aug 7 [cited 2018 Oct 30];17. Available from: https://www.ncbi.nlm.nih.gov/pmc/articles/PMC5545835/

19. Nicol E, Bradshaw D, Uwimana-Nicol J, Dudley L. Perceptions about data-informed decisions: an assessment of information-use in high HIV-prevalence settings in South Africa. BMC Health Serv Res [Internet]. 2017 Nov [cited 2018 Aug 13];17(S2). Available from: https://bmchealthservres.biomedcentral.com/articles/10.1186/s12913-017-2641-1

20. Dagnew E, Woreta SA, Shiferaw AM. Routine health information utilization and associated factors among health care professionals working at public health institution in North Gondar, Northwest Ethiopia. BMC Health Serv Res [Internet]. 2018 Sep 4 [cited 2018 Oct 30];18. Available from: https://www.ncbi.nlm.nih.gov/pmc/articles/PMC6122568/

21. Aqil A, Lippeveld T, Hozumi D. PRISM framework: a paradigm shift for designing, strengthening and evaluating routine health information systems. Health Policy Plan. 2009 May 1;24(3):217–28.

22. Nutley T, Gnassou L, Traore M, Bosso AE, Mullen S. Moving data off the shelf and into action: an intervention to improve data-informed decision making in Côte d'Ivoire. Glob Health Action [Internet]. 2014 Oct 1 [cited 2018 Oct 30];7. Available from: https://www.ncbi.nlm.nih.gov/pmc/articles/PMC4185136/

23. Tuti T, Bitok M, Paton C, Makone B, Malla L, Muinga N, et al. Innovating to enhance clinical data management using non-commercial and open source solutions across a

multi-center network supporting inpatient pediatric care and research in Kenya. J Am Med Inform Assoc JAMIA. 2016 Jan;23(1):184–92.

24. Wagenaar BH, Gimbel S, Hoek R, Pfeiffer J, Michel C, Manuel JL, et al. Effects of a health information system data quality intervention on concordance in Mozambique: time-series analyses from 2009–2012. Popul Health Metr [Internet]. 2015 Mar 26 [cited 2019 Jan 27];13. Available from: https://www.ncbi.nlm.nih.gov/pmc/articles/PMC4377037/

25. Mutale W, Chintu N, Amoroso C, Awoonor-Williams K, Phillips J, Baynes C, et al. Improving health information systems for decision making across five sub-Saharan African countries: Implementation strategies from the African Health Initiative. BMC Health Serv Res. 2013;1:S9.

26. Ahmed T, Lucas H, Khan AS, Islam R, Bhuiya A, Iqbal M. eHealth and mHealth initiatives in Bangladesh: A scoping study. BMC Health Serv Res. 2014 Jun 16;14:260.

27. WHO | Data Quality Review (DQR) Toolkit [Internet]. WHO. [cited 2018 Oct 23]. Available from: http://www.who.int/healthinfo/tools_data_analysis/dqr_modules/en/

28. Routine Data Quality Assessment Tool - User Manual — MEASURE Evaluation [Internet]. [cited 2019 Jan 30]. Available from: https://www.measureevaluation.org/resources/publications/ms-17-117

29. Maokola W, Willey BA, Shirima K, Chemba M, Schellenberg JRMA, Mshinda H, et al. Enhancing the routine health information system in rural southern Tanzania: successes, challenges and lessons learned. Trop Med Int Health. 2011;16(6):721–30.

30. Braa J, Heywood A, Sahay S. Improving quality and use of data through data-use workshops: Zanzibar, United Republic of Tanzania. Bull World Health Organ. 2012 May;90(5):379–84.

31. Monda J, Keipeer J, M.c W. Data integrity module for data quality assurance within an e-health system in sub-Saharan Africa. J E-Health. 2012 Feb;

32. Mpofu M, Semo B, Grignon J, Lebelonyane R, Ludick S, Matshediso E, et al. Strengthening monitoring and evaluation (M&E) and building sustainable health information systems in resource limited countries: lessons learned from an M&E task-shifting initiative in Botswana. BMC Public Health. 2014 Oct 3;14:1032.

33. Tuti T, Bitok M, Malla L, Paton C, Muinga N, Gathara D, et al. Improving documentation of clinical care within a clinical information network: an essential initial step in efforts to understand and improve care in Kenyan hospitals. BMJ Glob Health. 2016;1(1).

34. Gimbel S, Mwanza M, Nisingizwe MP, Michel C, Hirschhorn L. Improving data quality across 3 sub-Saharan African countries using the Consolidated Framework for Implementation Research (CFIR): results from the African Health Initiative. (Implementation science as an essential driver for sustainable health systems strengthening interventions: Lessons learned across the five-country African Health Initiative.). BMC Health Serv Res. 2017;

35. Randriamiarana R, Raminosoa G, Vonjitsara N, Randrianasolo R, Rasamoelina H, Razafimandimby H, et al. Evaluation of the reinforced integrated disease surveillance and response strategy using short message service data transmission in two southern regions of Madagascar, 2014-15. BMC Health Serv Res. 2018 Apr;18(1):265.

36. Ndira SP, Rosenberger KD, Wetter T. Assessment of Data Quality of and Staff Satisfaction with an Electronic Health Record System in a Developing Country (Uganda) A Qualitative and Quantitative Comparative Study. Methods Inf Med. 2008;47(6):489–98.

37. Wagenaar BH, Hirschhorn LR, Henley C, Gremu A, Sindano N, Chilengi R. Data-driven quality improvement in low-and middle-income country health systems: lessons from seven years of implementation experience across Mozambique, Rwanda, and Zambia. BMC Health Serv Res. 2017 Dec 21;17:830.

38. Nakibuuka J, Semwanga AR, Were MC. Implementation of USSD Technology to Improve Quality of Routinely Reported Health Data in a Resource-Limited Setting. Stud Health Technol Inform. 2019 Jul;1:162–5.

39. O'Connor EC, Hutain J, Christensen M, Kamara MS, Conteh A, Sarriot E, et al. Piloting a participatory, community-based health information system for strengthening community- based health services: findings of a cluster-randomized controlled trial in the slums of Freetown, Sierra Leone. J Glob Health. 2019 Jun;9(1).

40. Njuguna C, Jambai A, Chimbaru A, Nordstrom A, Conteh R, Latt A, et al. Revitalization of integrated disease surveillance and response in Sierra Leone post Ebola virus disease outbreak. BMC PUBLIC Health. 2019 Apr 2;19.

41. Yourkavitch J, Prosnitz D, Herrera S. Data quality assessments stimulate improvements to health management information systems: evidence from five African countries. J Glob Health [Internet]. [cited 2020 Mar 3];9(1). Available from: https://www.ncbi.nlm.nih.gov/pmc/articles/PMC6594668/

42. Ismail S, Alshmari M, Latif K, Ahmad HF. A Granular Ontology Model for Maternal and Child Health Information System. J Healthc Eng. 2017;9519321.

43. Kaushik A, Raman A. The new data-driven enterprise architecture for e-healthcare: Lessons from the Indian public sector. Gov Inf Q. 2015 Jan;32(1):63–74.

44. Lazzerini M, Senanayake H, Mohamed R, Kaluarachchi A, Fernando R, Sakalasuriya A, et al. Implementation of an individual patient prospective database of hospital births in Sri Lanka and its use for improving quality of care. BMJ OPEN. 2019 Jun;9(2).

45. Dombrowski JG, Ataide R, Marchesini P, Souza RM de, Marinho CRF. Effectiveness of the Live Births Information System in the Far-Western Brazilian Amazon. Cienc Saude Coletiva. 2015;20(4):1245–54.

46. Puttkammer N, Pettersen K, Hyppolite N, France G, Valles JS, Honore JG, et al. Identifying priorities for data quality improvement within Haiti's iSante EMR system: Comparing two methods. Health Policy Technol. 2017 Mar;6(1):93–104.

47. Trumbo SP, Contreras M, Garcia AGF, Escobar Diaz FA, Gomez M, Carrion V, et al. Improving immunization data quality in Peru and Mexico: two case studies highlighting challenges and lessons learned. Vaccine. 2018;36(50):7674–81.

48. Gaumer G, Hassan N, Murphy M. A simple primary care information system featuring feedback to clinicians. Int J Health Plann Manage. 2008 Sep;23(3):185–202.

49. Braa J, Heywood A, Sahay S. Improving quality and use of data through data-use workshops: Zanzibar, United Republic of Tanzania. Bull World Health Organ. 2012 May;90(5):379–84.

50. Nutley T, McNabb S, Salentine S. Impact of a decision-support tool on decision making at the district level in Kenya. Health Res Policy Syst. 2013 Sep 8;11:34.

52. Rajan D, Kalambay H, Mossoko M, Kwete D, Bulakali J, Lokonga J-P, et al. Health service planning contributes to policy dialogue around strengthening district health systems: an example from DR Congo 2008-2013. BMC Health Serv Res. 2014 Oct 31;14:522.

53. Landis-Lewis Z, Manjomo R, Gadabu OJ, Kam M, Simwaka BN, Zickmund SL, et al. Barriers to using eHealth data for clinical performance feedback in Malawi: A case study. Int J Med Inf. 2015 Oct;84(10):868–75.

54. Uneke CJ, Langlois EV, Uro-Chukwu HC, Chukwu J, Ghaffar A. Fostering access to and use of contextualised knowledge to support health policy-making: lessons from the Policy Information Platform in Nigeria. Health Res Policy Syst. 2019 Apr 8;17:38.

55. La Vincente S, Aldaba B, Firth S, Kraft A, Jimenez-Soto E, Clark A. Supporting local planning and budgeting for maternal, neonatal and child health in the Philippines. Health Res Policy Syst. 2013 Jan 23;11:3.

56. Hosseini M, Ahmadi M, Dixon BE. A Service Oriented Architecture Approach to Achieve Interoperability between Immunization Information Systems in Iran. AMIA. 2014;1:1797–805.

57. Beane A, De Silva AP, Athapattu PL, Jayasinghe S, Abayadeera AU, Wijerathne M, et al. Addressing the information deficit in global health: lessons from a digital acute care platform in Sri Lanka. BMJ Glob Health. 2019 Feb;4(1).

58. Matheson AI, Baseman JG, Wagner SH, O'Malley GE, Puttkammer NH, Emmanuel E, et al. Implementation and expansion of an electronic medical record for HIV care and treatment in Haiti: An assessment of system use and the impact of large-scale disruptions. Int J Med Inf. 2012 Apr;81(4):244–56.

59. Ghosh A, McCarthy S, Halcomb E. Perceptions of primary care staff on a regional data quality intervention in Australian general practice: a qualitative study. BMC Fam Pract. 1471st–2296th ed. 2016 Apr;

60. McMurray J, Zhu L, McKillop I, Chen H. Ontological modeling of electronic health information exchange. J Biomed Inform. 2015 Aug;

61. Lucyk K, Tang K, Quan H. Barriers to data quality resulting from the process of coding health information to administrative data: a qualitative study. BMC Health Serv Res. 1472nd–6963rd ed. 2017 Nov;

62. Ben-Assuli O, Shabtai I, Leshno M. Using electronic health record systems to optimize admission decisions: the Creatinine case study. Health Informatics J. 1741st–2811th ed. 2015 Mar;

63. Snowden A, Kolb H. Two years of unintended consequences: introducing an electronic health record system in a hospice in Scotland. J Clin Nurs. 2017 May;26(9–10):1414–27.

64. Del Fiol G, Huser V, Strasberg HR, Maviglia SM, Curtis C, Cimino JJ. Implementations of the HL7 Context-Aware Knowledge Retrieval ("Infobutton") Standard: challenges, strengths, limitations, and uptake. J Biomed Inform. 2012 Aug;45(4):726–35.

65. Wilkinson DI, McCarthy M. Use of comparative data for integrated cancer services. BMC Health Serv Res. 1472nd–6963rd ed. 2007;

Supporting Information

S1A Box. Combinations of search terms formulated in Ovid for studies on data quality interventions

S1B Box. Combinations of search terms formulated in Ovid for studies on data use interventions

S1C Box. Contents of the data charting format used

S2A Table. Characteristics of studies on data quality interventions in low- and middle-income countries 2008-2020

S2B Table. Characteristics of studies on data use interventions in low- and middle-income countries 2008-2020

S3 File. Protocol: Improving Quality of Routine Health Information System and Data Use in Low- and Middle-Income Countries: A Scoping Review

S4 file. Preferred Reporting Items for Systematic reviews and Meta-Analyses extension for Scoping Reviews (PRISMA-ScR) Checklist

Click here to access/download;Figure;PublicationFiles-figure_1_2_final_10_04_20.docx

Figure 1: The Process and Results of Literature Search for Data Quality

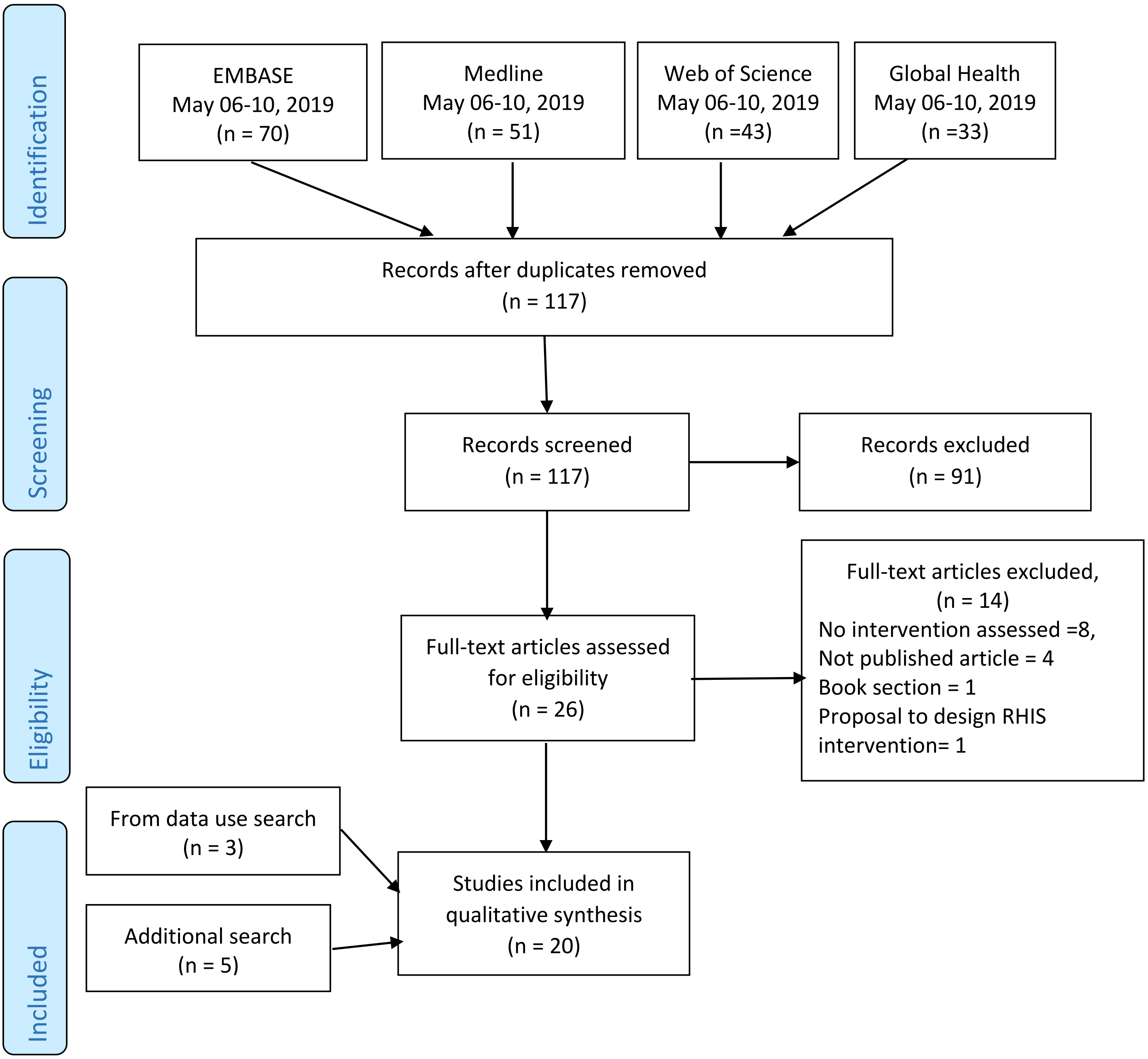

Figure 2: The Process and Results of Literature Search for Data Use

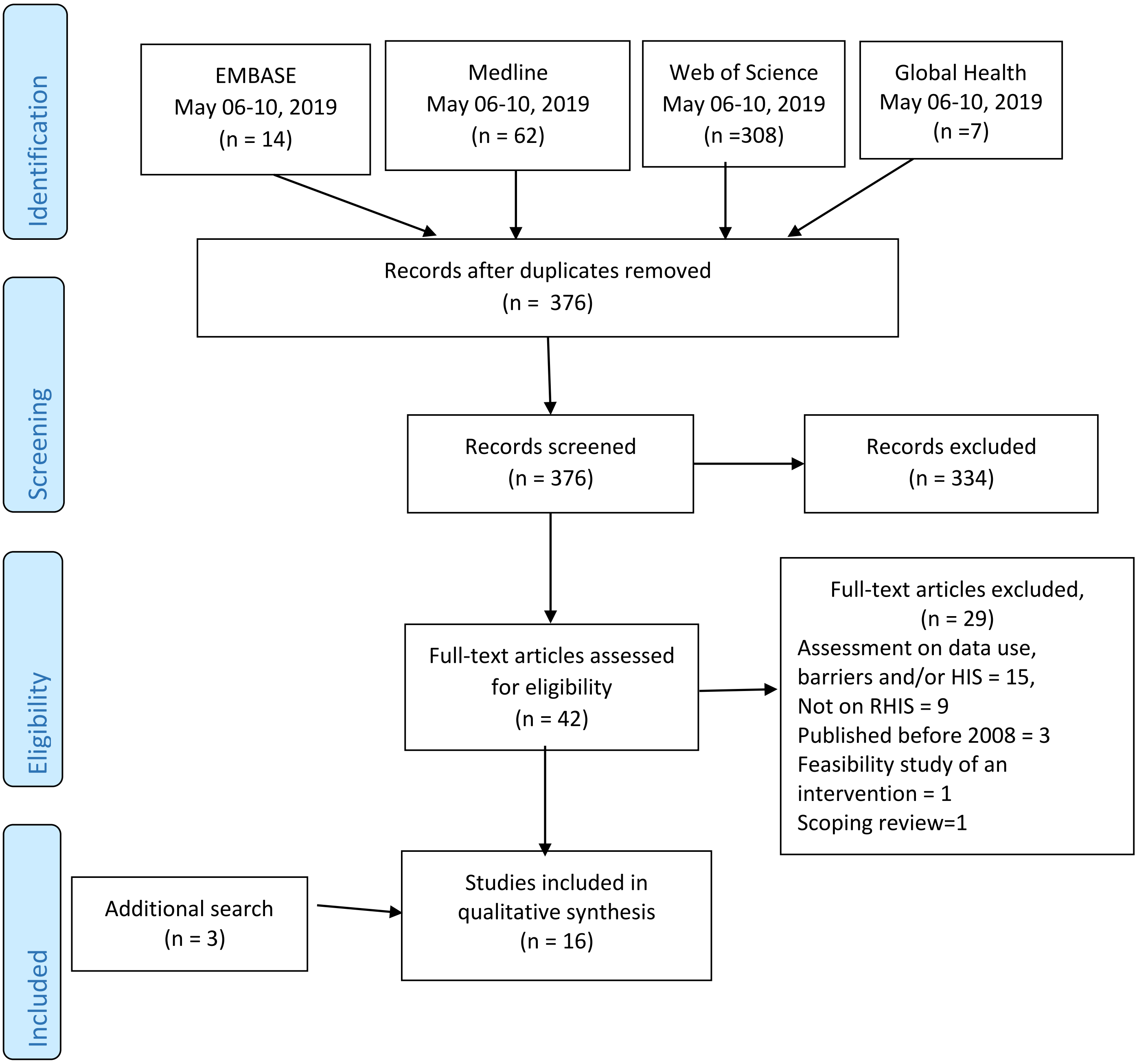

Click here to access/download
Supporting Information
PublicationFiles-s1a_s1c_boxes_final.docx

Click here to access/download
Supporting Information
PublicationFiles-s2a_s2b_tables_final_15_04_2020.docx

Click here to access/download
Supporting Information
PublicationFiles-s3_file_protocol_final.docx

Click here to access/download
Supporting Information
PublicationFiles-s4_file_prisma-scr_fillable_checklist_done.docx
